# Mountains of diversity: a systematic revision of the Andean rodent genus *Oreoryzomys* (Cricetidae: Sigmodontinae)

**DOI:** 10.7717/peerj.20515

**Published:** 2026-01-09

**Authors:** Jorge Brito, Rocío Vargas, Nicolás Tinoco, Rubí García, Julio C. Carrión-Olmedo, Claudia Koch, Ricarda Wistuba, Carlos Nivelo-Villavicencio, Ulyses F.J. Pardiñas

**Affiliations:** 1 Instituto Nacional de Biodiversidad (INABIO), Quito, Ecuador; 2 Instituto de Diversidad y Evolución Austral (IDEAUS-CONICET), Puerto Madryn, Chubut, Argentina; 3 Fundación Cóndor Andino, Quito, Ecuador; 4 Dosel Ecuador, Quito, Ecuador; 5 Sección de Mastozoología, Museo de Zoología, Facultad de Ciencias Exactas y Naturales, Pontificia Universidad Católica del Ecuador, Quito, Ecuador; 6 Leibniz Institute for the Analysis of Biodiversity Change/Museum Koenig, Bonn, Germany

**Keywords:** Andes, Ecuador, New species, *Oreoryzomys balneator*, *Oreoryzomys hesperus*, Oryzomyini, Peru

## Abstract

The until recently monotypic cricetid genus *Oreoryzomys* inhabits piedmont and cloud forests, primarily in eastern Ecuador and northwestern Peru. Erected following the taxonomic revision of a polytypic *Oryzomys* complex two decades ago, *Oreoryzomys* has remained poorly understood, with most references limited to the original descriptions of its type species (*O. balneator*) and a subspecies (*O. b. hesperus*). Here, we present an integrative taxonomic revision of the genus, based on new field collections and comprehensive museum-based analyses. Phylogenetic reconstructions from mitochondrial and nuclear gene sequences, combined with morphometric and qualitative morphological data, support the recognition of three species: (1) a redescribed *O. balneator* from central-eastern Ecuador; (2) *O. hesperus*, elevated to full species rank based on topotypic material; and (3) a new species from populations of the Quijos River Valley, northeastern Ecuador. This revision triples the known species diversity of *Oreoryzomys* and highlights the genus as a notable radiation of small-bodied oryzomyines adapted to Andean environments. Our findings emphasize the need for systematic revisions of other poorly known Andean rodents to better reveal the hidden diversity of cricetids and the role of the Andes in shaping Neotropical biodiversity.

## Introduction

The Tropical Andes have emerged as a paradigmatic hotspot of Neotropical biodiversity, thanks to numerous studies conducted under the framework of integrative taxonomy over the past quarter-century (*e.g.*, Myers et al., 2000; Pérez-Escobar et al., 2019; Bax & Francesconi, 2019; Comer et al., 2022; Yánez-Muñoz et al., 2024). Among mammals, cricetid rodents have not been exempt from this wave of taxonomic revision, driven primarily by extensive fieldwork and intensive genetic analyses. In fact, within the notably diverse subfamily Sigmodontinae, the tribe Oryzomyini stands out as the most speciose. It reaches a noticeable species richness in the Tropical Andes, a region that can also represent its plausible center of original diversification (*e.g.*, Reig, 1986; Valencia-Pacheco et al., 2011; Prado et al., 2015; Percequillo et al., 2021).

Focusing on Ecuador—one of the countries most profoundly shaped by the Andes—recent research has revealed that current estimates of oryzomyine diversity are clearly limited. Over the past five years, a new genus, *Pattonimus*, has been described from the northern montane forests, comprising at least three species (Brito et al., 2020). Additionally, several other new species have been identified, including one belonging to the rare arboreal genus *Mindomys* (Brito et al., 2020; Brito et al., 2021a; Brito et al., 2021b; Brito et al., 2025; Tinoco et al., 2023).

*Oreoryzomys* is one of several genera that arose from the taxonomic reorganization of the traditional “wastebasket” genus *Oryzomys* (Weksler, Percequillo & Voss, 2006; D’Elía & Pardiñas, 2007). Despite being one of ten new oryzomyine genera recognized in that revision, *Oreoryzomys* remains uniquely understudied. The other genera—*Aegialomys, Cerradomys, Eremoryzomys, Euryoryzomys, Hylaeamys, Mindomys, Nephelomys, Sooretamys*, and *Transandinomys*—have all been the subject of contemporary systematic and phylogenetic research (*e.g.*, Brennand, Langguth & Percequillo, 2013; Chiquito, D’elia & Percequillo, 2014; Prado & Percequillo, 2016; Uturunco & Pacheco, 2016; Guilardi et al., 2020; Brito et al., 2022b; Brito et al., 2022a; Di-Nizo, Suárez-Villota & Silva, 2022; Ruelas et al., 2021).

In the nearly one hundred years since its original description, *Oreoryzomys* has received limited scientific attention. After the brief original account by Thomas (1900), *Oryzomys balneator hesperus* was described by Anthony (1924), who documented its presence on the western slopes of the Andes. No significant taxonomic developments followed until Weksler, Percequillo & Voss (2006) elevated these small rodents to a new genus—*Oreoryzomys*—a name derived from the Greek word *oros*, meaning “mountain,” reflecting their Andean origins. More recently, Percequillo (2015:439) reviewed the limited information available on *O. balneator*, remarking that “the validity of *Oryzomys balneator hesperus* Anthony remains to be determined…Until that definition has been achieved, I provisionally recognize Anthony’s name as a synonym of *Oreoryzomys balneator*.”

Currently, *Oreoryzomys* is considered a monotypic genus, with *O. balneator* inhabiting montane rainforests of the Andes in Ecuador and northern Peru. The species is known from only a handful of specimens (Percequillo, 2015). Remarkably, *Oreoryzomys* remains the only extant sigmodontine genus for which no published photographs of the skull or dentition exist—a striking reflection of how little is known about this lineage.

Through a series of intensive field expeditions aimed at surveying the mammalian diversity of Ecuador, combined with the examination of specimens housed in scientific collections, we assembled a representative sample of *Oreoryzomys*. By integrating multiple methodological approaches—including both qualitative and quantitative morphological analyses—supported by genetic data from the mitochondrial gene Cytb and the nuclear gene IRBP, we conducted a comprehensive revision of the genus’s alpha taxonomy. Our results lead us to describe a new species from northeastern Ecuador and to elevate *hesperus* to full species status. Additionally, we provide a revised generic diagnosis of *Oreoryzomys*, incorporating these taxonomic updates along with novel anatomical data (*e.g.*, stomach morphology; postcranial skeleton) presented herein.

## Materials & Methods

### Studied specimens

A qualitative morphological analysis of 78 specimens belonging to the genus *Oreoryzomys* in Ecuador was conducted. The majority of the Ecuadorian specimens analyzed were obtained by the senior author and collaborators during recent field expeditions in the Cordillera de Kutukú, Sangay National Park, the Cordillera de Chilla, the Llanganates-Sangay Corridor, and Yanayacu. These surveys encompassed a total trapping effort of 12,800 trap/nights. Specimen capture, handling, and preservation procedures adhered to the guidelines set forth by the American Society of Mammalogists (Sikes, Care & Use Committee of the American Society of Mammalogists, 2016). The collection permits issued by Ministerio del Ambiente, Agua y Transición Ecológica del Ecuador (MAATE) that allowed the study to be carried out are as follows: No. 005-2014-I-B- DPMS/MAE, 007-IC-DPACH-MAE-2016, 005-ICFLOFAU-DPAEO-MAE, 003-2019-IC-FLO-FAU-DPAC/MAE, MAE-DNB-CM-2019-0126, MAAE-ARSFC-2020-0642, MAAE-ARSFC-2021-1644MAATE-ARSFC-2023-0145, MAATE-ARSFC-2024-1064, and the authorization for access to genetic resources No. MAATE-DBI-CM-2023-0334. The collected material was compared with specimens housed in the mammal collections of the following Ecuadorean institutions: Instituto Nacional de Biodiversidad (INABIO; Quito), formerly known as Museo Ecuatoriano de Ciencias Naturales (MECN); Museo de la Escuela Politécnica Nacional (MEPN; Quito); and Museo de Zoología de la Pontificia Universidad Católica del Ecuador (QCAZ; Quito).

### Anatomy, age criteria, and measurements

To describe cranial anatomy, we followed the criteria and nomenclature established by Hershkovitz (1962), Voss (1988), Carleton & Musser (1989), Steppan (1995) and Wible & Shelley (2020). Molar occlusal morphology was assessed based on Reig (1977). Regarding soft anatomy, palate was described according to Quay (1954), rhinarium morphology according to Pardiñas et al. (2022), digestive system according to Carleton (1973), and female genitalia (Hooper & Musser, 1964; Rodriguez et al., 2011; Cunha et al., 2020). We followed the terminology and definitions employed by Tribe (1996) and Costa et al. (2011) for age classes and restricted the term “adults” for those in categories 3 and 4. We obtained the following external measurements in millimeters (mm), some of them registered in the field and reported from specimen tags, others recorded in museum cabinets: head and body length (HBL), tail length (TL), hind foot length with claw (HF), ear length (E), and body mass (W) in grams. Cranial measurements were obtained with digital calipers, to the nearest 0.01 mm. We employed the following dimensions (see Voss, 1988; Brandt & Pessôa, 1994; Musser et al., 1998 for descriptions and illustrations): occipitonasal length (ONL), condylo-incisive length (CIL), length of upper diastema (LD), crown length of maxillary toothrow (LM), length of incisive foramen (LIF), breadth of incisive foramina (BIF), length of nasals (LN), breadth of rostrum (BR), length of palatal bridge (LPB), breadth of bony palate (BBP), least interorbital breadth (LIB), zygomatic breadth (ZB), breadth of zygomatic plate (BZP), orbital fossa length (OFL), bular breadth (BL), length of mandible (LJ), crown length of mandibular toothrow (LMI), and length of inferior diastema (LDI). Finally, dental measurements, the maximum length and width of each individual molar, were obtained under magnification using a reticulate eyepiece: length of M1 (LM1), width of M1 (WM1), length of M2 (LM2), width of M2 (WM2), length of M3 (LM3), width of M3 (WM3), length of m1 (Lm1), width of m1 (Wm1), length of m2 (Lm2), width of m2 (Wm2), length of m3 (Lm3), width of m3 (Wm3).

### Scanning

To enable detailed morphological analyses of cranial structures, we performed high-resolution micro-computed tomography (micro-CT) scans of selected specimens, including two specimens of *O. balneator* (MECN 5009, 5795), one specimen of *O. balneator hesperus* (MECN 4789), and one specimen of a newly described species (MECN 8278). Scanning was carried out at the Leibniz Institute for the Analysis of Biodiversity Change/Museum Koenig (LIB, Bonn, Germany) using a Bruker SkyScan 1173 desktop micro-CT system (Bruker MicroCT, Kontich, Belgium).

To ensure maximum stability during scanning, the skulls were fixed in cotton wool and placed within small plastic containers. Scan settings included a source voltage of 43 kV and a current of 116 µA without filter use. Exposure times were set to 500 ms per projection, with a total of 800 projections acquired, applying frame averaging value of 4. Rotation was continuous over 180°, at step sizes of 0.3°, resulting in total scan time of about 46 min and an isotropic voxel size of 17.03 µm. Reconstructions were generated using N-Recon software (v1.7.1.6).

The skulls and mandibles were digitally separated using Amira software (Thermo Fisher Scientific, Hillsboro, OR, USA) and all datasets were rendered into 3D with Amira or CTVox (v3.0.0 r1114, Bruker MicroCT).

### DNA amplification and sequencing

We used tissue samples (liver or muscle) from specimens deposited in QCAZ and INABIO (Table 1). The tissues were originally preserved in 90% ethanol and stored in an ultra-freezer at −80 °C. The DNA extraction was performed using a guanidine thiocyanate protocol.

**Table 1:** Species, vouchers, and GenBank accession numbers for newly generated DNA sequences used in genetic analyses. The sequences generated in this work are shown in bold. The MECN sequences were obtained with Oxford Nanopore, while the QCAZ were obtained by Macrogen.

**Species**	**Voucher ID**	**Cytb**	**IRPB**
*Microryzomys altissimus*	QCAZ 8353	EU579502	EU649055
	–	EU258535	
*Microryzomys minutus*	n/a	EU58387	
	n/a	AF108698	
	n/a		AY163592
*Oreoryzomys balneator*	MECN 6343	PV962886	
	MECN 6345	PV962887	
	MECN 7242	PV962888	
	MECN 5840	PV962897	
	MECN 5839	PV962891	
	QCAZ 17484	PV962889	
	QCAZ 17575	PV962890	
	QCAZ17576	PV962892	
	QCAZ17479	PV962893	PV962868
	QCAZ17572	PV962894	PV962864
	QCAZ 17569	PV962895	PV962866
	QCAZ 17574	PV962896	PV962863
	QCAZ 17480	PV962898	
	QCAZ 17486	PV962899	PV962867
	QCAZ 17573	PV962900	PV962865
*Oreoryzomys hesperus*	MECN 4789	PV962883	
	QCAZ 13196	PV962884	
	QCAZ 13280	PV962885	
	XX	GU126535	
	XX		AY163617
	AMNH 268144	EU579510	
*Oreoryzomys jumandi* sp. nov.	MECN 8279	PV962869	
	MECN 8277	PV962870	
	MECN 8278	PV962871	
	QCAZ 7657	PV962872	PV962861
	QCAZ 7738	PV962873	
	QCAZ 15922		PV962858
	QCAZ 15927	PV962874	PV962857
	QCAZ 15926		PV962859
	QCAZ 7700	PV962875	
	QCAZ 7756	PV962876	
	QCAZ 7798	PV962877	
	QCAZ 15924	PV962878	
	QCAZ 7787	PV962879	
	QCAZ 4993	PV962880	PV962862
	QCAZ 7676	PV962881	PV962860
	QCAZ 7677	PV962882	
	QCAZ 7838	EU258534	EU649068

We selected two genes for analyses: the mitochondrial cytochrome b (Cytb) and the nuclear interphotoreceptor retinoid-binding protein (IRBP). For Cytb, samples from QCAZ were amplified using the following combinations of primers: MVZ05 with MVZ16, and MVZ05 with MVZ14, following the PCR protocols described by Smith & Patton (1993) and Bonvicino & Moreira (2001). For IRBP, we used the primers A1 and F1 and followed the protocol outlined in D’Elía et al. (2006). The samples of INABIO (Table 1) were sequenced using Oxford Nanopore at the Laboratorio de Secuenciación de Ácidos Nucleicos of the Instituto Nacional de Biodiversidad (INABIO) in Quito, Ecuador under the methodology described in Carrión-Olmedo & Brito (2025). The amplicons from QCAZ samples (QCAZ 7657, 7738, 7700, 7798, 15924, 7756, 7787, 7677, 15927, 13196, 13280, 8334, 17575, 17548, 17576, 17480, 17573, 17486, 17574, 17569, 17572, 17479) were sent to Macrogen, South Korea, for sequencing. The resulting sequences were edited and assembled using Geneious R11 (https://www.geneious.com/), during the editing process, the ends of some sequences were removed; this was done when the ends were of poor quality after assembly.

### Phylogenetic analyses

We conducted phylogenetic analyses using two datasets: one consisting of Cytb sequences and the other of IRBP sequences. To assess the monophyly of the genus *Oreoryzomys*, both datasets included sequences from closely related genera, including *Microryzomys*, *Neacomys*, and *Oligoryzomys* (*i.e.,* the clade C of Oryzomyini; see Weksler, 2006; Brito et al., 2020). The sequences generated and obtained from the genbank were aligned using the ClustalW tool (Thompson, Higgins & Gibson, 1994) in the Geneious R11 program (https://www.geneious.com). We used PartitionFinder2 (Lanfear et al., 2017) to determine the best partitioning scheme and substitution models. The Cytb alignment was divided into three partitions with the following models: first position (GTR+I+G), second position (HKY+I+G), and third position (GTR+I+G). The IRBP gene was also separated in three partitions, and the best model for each partition was K80+G.

Bayesian analyses were performed using Markov Chain Monte Carlo (MCMC) sampling as implemented in MrBayes 3.2.6 (Ronquist et al., 2012). Uniform interval priors were assumed for all parameters except base composition, for which a Dirichlet prior was used. We ran four independent analyses, each with 10 million generations, two heated chains, and sampling of trees and parameters every 10,000 generations. We use a burn-in of 0.25 to discard some part of trees and the remaining were used to estimate posterior probabilities for each node. To evaluate the convergence we use the command *sump* and *sumt* in Mr. Bayes, we evaluate the effective sample size (ESS≥100) and verifying the potential scale reduction factor (PSRF = 1). The analyses was conducted on the CIPRES Science Gateway (Miller, Pfeiffer & Schwartz, 2010).

Maximum-likelihood analyses were conducted using IQ-TREE web server (http://iqtree.cibiv.univie.ac.at). Nodal support was assessed with 1,000 ultrafast bootstrap replicates (UFboot; Minh, Nguyen & Von Haeseler, 2013) implemented in IQ-TREE v2 (Minh et al., 2020). The resulting consensus tree was derived from these replicates, and nodes with UFboot values exceeding 95% were considered to indicate strong support.

The genetic distances (p-distances) were calculated in MEGA X (Kumar et al., 2018) to corroborate the divergences observed in the phylogenetic trees.

### Morphology

The craniodental measurements of the 78 analyzed specimens of *Oreoryzomys* were compiled in a single matrix totalizing 2,396 values. The morphometric analyses were conducted using the MorphoTools2 package in R. Data were initially loaded from a CSV file and cleaned using the janitor package to ensure consistent column names and conversion of empty values in numeric columns to NA. Categorical variables, such as ID, population, and taxon, were transformed into factors, while quantitative variables were isolated to form the core of the morphometric dataset.

The morphodata object was created as a structured list containing sample identifiers, populations, taxa, and numeric variables. This object allowed for exploratory analysis using specific functions such as summary, samples, and characters. Data normality was assessed through the Shapiro–Wilk test, and the results were exported for further evaluation.

Multivariate analyses, including Principal Component Analysis (PCA), were performed to identify patterns of morphometric variation, visualized through two- and three-dimensional plots. Additionally, Canonical Discriminant Analysis (CDA) were conducted to evaluate clustering among taxa and populations, complemented by confidence ellipse plots and biplots. Missing values were imputed using k-Nearest Neighbors imputation to minimize their impact on the analyses. This methodology integrated multiple approaches for a robust description of morphometric patterns.

### New zoological taxonomic names

The electronic version of this article in Portable Document Format (PDF) will represent a published work according to the International Commission on Zoological Nomenclature (ICZN), and hence the new names contained in the electronic version are effectively published under that code from the electronic edition alone. This published work and the nomenclatural acts it contains have been registered in ZooBank, the online registration system for the ICZN. The ZooBank LSIDs (Life Science Identifiers) can be resolved and the associated information viewed through any standard web browser by appending the LSID to the prefix http://zoobank.org/. The LSID for this publication is: urn:lsid:zoobank.org:pub:5E61A4AC-BF4E-413C-B2D6-E9139759D6E3. The online version of this work is archived and available from the following digital repositories: PeerJ, PubMed Central and CLOCKSS.

## Results

### Phylogenetic analyses

A total of 33 Cytb sequences, ranging from 612 to 1,140 bp, were generated, along with 12 IRBP sequences, ranging from 601 to 786 bp. The final mitochondrial Cytb matrix included 68 terminals (taxa) with lengths of 567 to 1,140 bp, while the nuclear IRBP matrix included 29 terminals (taxa) with lengths of 601 to 1,266 bp. The Cytb matrix contained 452 variable sites and 383 parsimony-informative sites, whereas the IRBP matrix contained 93 variable sites and 37 parsimony-informative sites.

**Figure 1:**
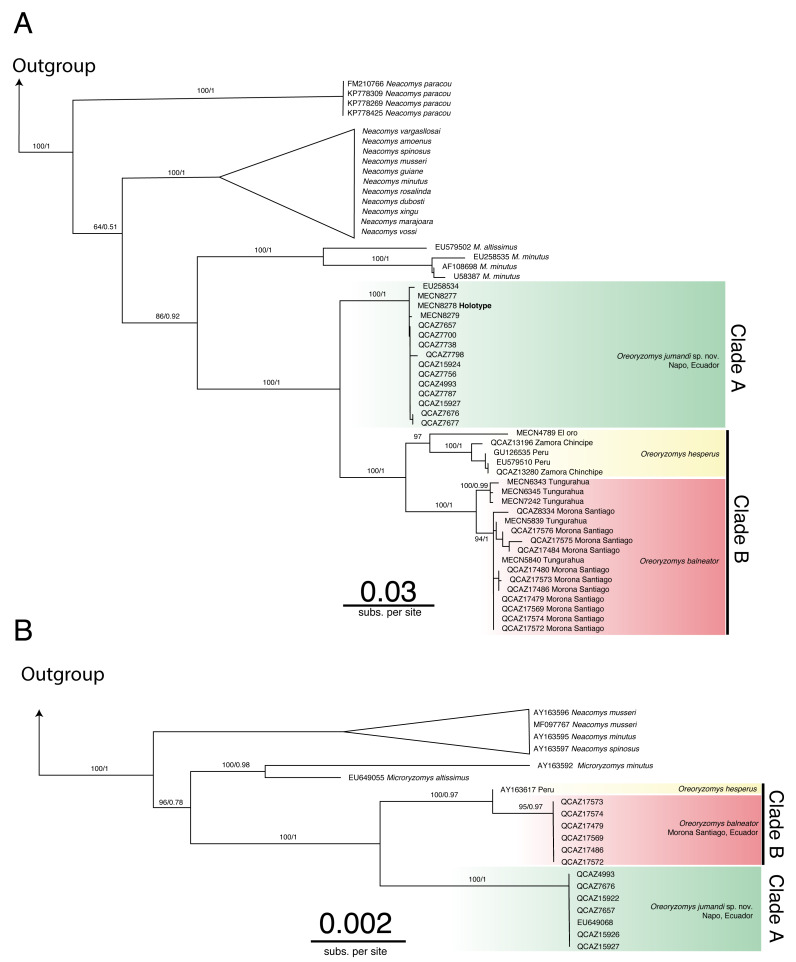
Phylogenetic trees generated through Maximum likelihood trees of *Oreoryzomys* based on Cytb (A) and IRBP (B) genes. Node values indicate ultrafast bootstrap support (%) and posterior probability (PP). Terminals marked with an asterisk (*) correspond to specimens from type localities.

The genus *Oreoryzomys* was recovered as monophyletic and as the sister genus to *Microryzomys* (Figs. 1A–1B; Figs. S1–S4). Within *Oreoryzomys*, two clearly differentiated clades with high statistical support (posterior probabilities/bootstrap values) were identified: clade “A” (Fig. 1A; 1.00/100) includes samples from northeastern Ecuador (Napo), while clade “B” (Fig. 1A; 1.00/100) groups samples from central Ecuador (Tungurahua), the southwestern and eastern Andean foothills of Ecuador (El Oro, Zamora Chinchipe, and Morona Santiago), and northern Peru (Cajamarca). Within this latter clade, sequences from specimens (MECN 6343, 6345, 7242) collected close to the type locality of *O. balneator* (*i.e.,* “Mirador, Baños, Tungurahua” Thomas, 1900), were included. Internally, this clade was resolved in two groups: one (1.00/100) includes samples from Tungurahua (type locality) and Morona Santiago, while the other group (0.97/0.99) includes samples from El Oro, Zamora Chinchipe and two samples from Cajamarca, Peru.

The results of Bayesian Inference (BI) and Maximum Likelihood **(**ML) based on IRBP presented similar topologies to those described for Cytb, forming two clearly differentiated clades “A” and “B” (Fig. 1B). Clade “A” (1.00/100) includes samples from northeastern Andean slopes of Ecuador (Napo), while clade “B” (1.00/0.97) encompasses samples from southeastern Andean foothills (Morona Santiago), as well as northern Peru (Cajamarca).

The genetic distance between the clades “A” and “B” was approximately 5.33% ± 0.53%, indicating considerable divergence (Table 2). Within clade “B”, the genetic distance between the group of samples from “El Oro/Zamora Chinchipe/Cajamarca” and the samples from Tungurahua/Morona Santiago was 3.83% ± 0.53%. The genetic distance values between clades “A” and “B” fall within the range reported for species of the sister genus *Microryzomys*: *M. altissimus* vs*. M. minutus*= 5–6% (Calvache, 2020), whereas within clade “B”, the distances between the two sample groups are comparable to those observed in species of closely related genera such *as Neacomys: N. carceleni* vs*. N. amoenus*= 3.8% (Tinoco et al., 2023)*, N. marajoara* and *N. xingu* = 3.9% (Semedo et al., 2021)*.*

**Table 2:** *p*-Distances among Oreoryzomys species based on mitochondrial Cytb sequences (500–1,140 bp). Values represent nucleotide differences, with the upper-right portion showing standard deviations. Lower values indicate higher genetic similarity, while higher values reflect greater evolutionary divergence.

	* *	**1**	**2**	**3**
**1.**	*Oreoryzomys balneator*	–	0.53%	0.71%
**2.**	*Oreoryzomys hesperus*	3.83%	–	0.63%
**3.**	*Oreoryzomys* sp. nov	6.41%	5.33%	–

The phylogenetic analyses and the genetic variation found suggest that the populations represented in clade “B” (El Oro/Zamora Chinchipe/Cajamarca) can be considered a taxonomic entity distinct from *Oreoryzomys balneator*. Although *O. balneator* exhibits intraspecific variation (Fig. 1B), the evidence obtained suggests that the samples corresponding to the El Oro and Zamora Chinchipe/Cajamarca clade may represent distinct taxonomic entities (Figs. 1A–1B).

### Morphology analyses

The analyzed matrix of morphometrical characters comprises 2,808 measurements with 78 missing values. Morphological quantitative revision revealed that Napo samples (considered here a new species of *Oreoryzomys*) are different from those of southern species. The first two components of the PCA explained 36.90% of the morphometrical variation with PC1 explaining 19.65% and PC2 17.25%; condyle-incisive length (CIL) and bular breadth (BL) contributed great variation respectively (Table 3).

**Table 3:** Loadings and percentage of the explained variation of the principal component analysis (first two principal components) and of the canonical discriminant analysis (first two discriminant functions) performed on three species of the genus of rodent *Oreoryzomys*. Acronyms of variables are explained in the main text (Materials and Methods section).

	**PC1 (19.65%)**	**PC2 (17.25%)**	**Can1 (69%)**	**Can2 (31%)**
HBL	0.122142282	0.016663685	−0.0140638	0.058127602
TL	0.112690515	−0.0297833	−0.049942073	0.153592727
HF	0.11318933	−0.23514596	0.385976033	0.559235146
E	0.154529321	−0.18579209	0.49401744	0.452261974
W	0.085127479	0.263513016	0.159910195	−0.851804671
ONL	0.256175294	−0.1623381	0.150891938	0.349576282
CIL	0.322323824	0.014859673	0.220027022	−0.050731109
LD	0.210632287	0.205585308	−0.006193855	−0.295395843
LM	0.104338969	0.109509063	−0.127616923	−0.136060242
LIF	0.130533495	0.211240062	−0.335019087	−0.23103201
BIF	0.172863886	0.120041907	0.216628425	−0.510113316
LN	0.175941503	0.092615607	0.138236683	−0.358246284
BR	0.221520541	0.005229914	0.211675986	0.002779457
LB	0.288297209	−0.01525366	0.36738785	0.081990906
LPB	0.026480649	0.172433419	−0.538730201	−0.172381352
BBP	−0.04307019	0.242354225	−0.613615232	−0.141036394
LIB	0.155760187	0.125076796	0.3943454	−0.37114466
ZB	0.321372007	−0.09636086	0.609177598	0.085438883
BZP	0.209819717	−0.00671225	0.444931201	0.078237148
OFL	0.301182383	−0.04910728	0.616502986	0.023691453
BL	−0.08167439	0.345752533	−0.334183055	−0.591093885
LJ	0.15905665	0.250487803	−0.182612142	−0.411741859
LMI	0.042702956	0.072241645	−0.166654136	−0.169063144
LDI	−0.03675801	0.333074475	−0.397629957	−0.441128243
LM1	0.14161359	−0.07579865	0.03067485	0.32336189
WM1	0.086890379	0.200518431	−0.037692452	−0.118502497
LM2	0.057199739	−0.26549596	0.135049332	0.591142756
WM2	0.139840929	0.251087919	0.046982363	−0.409532709
LM3	0.174871669	−0.07773981	0.401755725	0.169188235
WM3	0.14264339	−0.0229709	0.531618953	−0.000207725
Lm1	0.043191148	−0.15602891	0.001801861	0.427331112
Wm1	0.06695698	0.178173076	0.306227662	−0.485460563
Lm2	−0.00304837	−0.10144968	−0.194533815	0.247507458
Wm2	0.080935984	0.085868425	0.14587508	−0.119913158
Lm3	0.242774665	−0.11907285	0.594920053	0.174681743
Wm3	0.142920374	−0.05087593	0.655677861	−0.056125413

**Figure 2:**
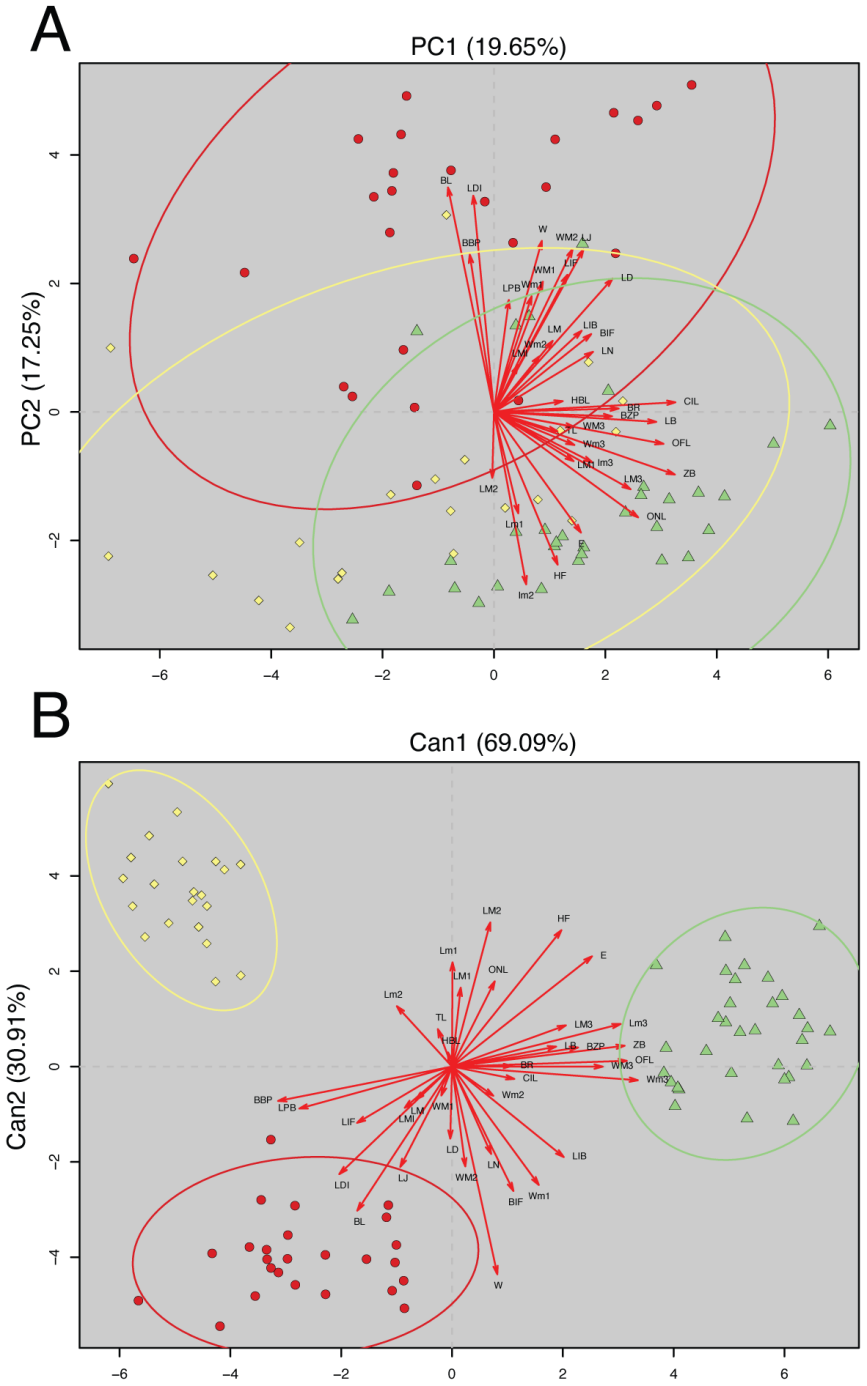
Principal Component Analysis (PCA) and Canonical Discriminant Analysis (CDA) of *Oreoryzomys* species. (A) PCA scatterplot showing individual distribution along the first two principal components. (B) CDA scatterplot illustrating group separation along the first two canonical axes. Ellipses indicate 95% confidence intervals for each species cluster. Red arrows denote variable contributions to each axis. 1). Clade Tungurahua + Morona Santiago (red circles), clade El Oro + Zamora Chinchipe (yellow diamonds) and clade Napo (green triangles). Variable codes are defined in the text.

In the PCA scatterplot (Fig. 2A), the three clades; (1) Tungurahua/Morona Santiago, (2) El Oro/Zamora Chinchipe and (3) Napo form partially overlapping but distinguishable clusters, with the Napo clade occupying a unique morphospace toward the positive end of PC1 and the lower half of PC2. Morphological traits such as CIL and BL contribute significantly to the observed variation, as indicated by the lengths and directions of the red vectors.

The CDA showed even stronger group separation (Fig. 2B). The first canonical axis (Can1) explained 69.09% of the variation, while the second axis (Can2) accounted for 30.91%. As illustrated in Fig. 2B, the three clades are clearly discriminated with no visible overlap among their 95% confidence ellipses. The Napo clade clusters tightly in the positive Can1 region, clearly distinct from El Oro/Zamora Chinchipe on the upper left quadrant and Tungurahua/Morona Santiago on the bottom left.

Overall, multivariate analyses support the metrical distinctiveness of the Napo clade. The consistency between PCA and CDA plots indicates robust differentiation among the three species based on craniodental and external morphometric traits.

### New anatomical and morphological data

**Figure 3:**
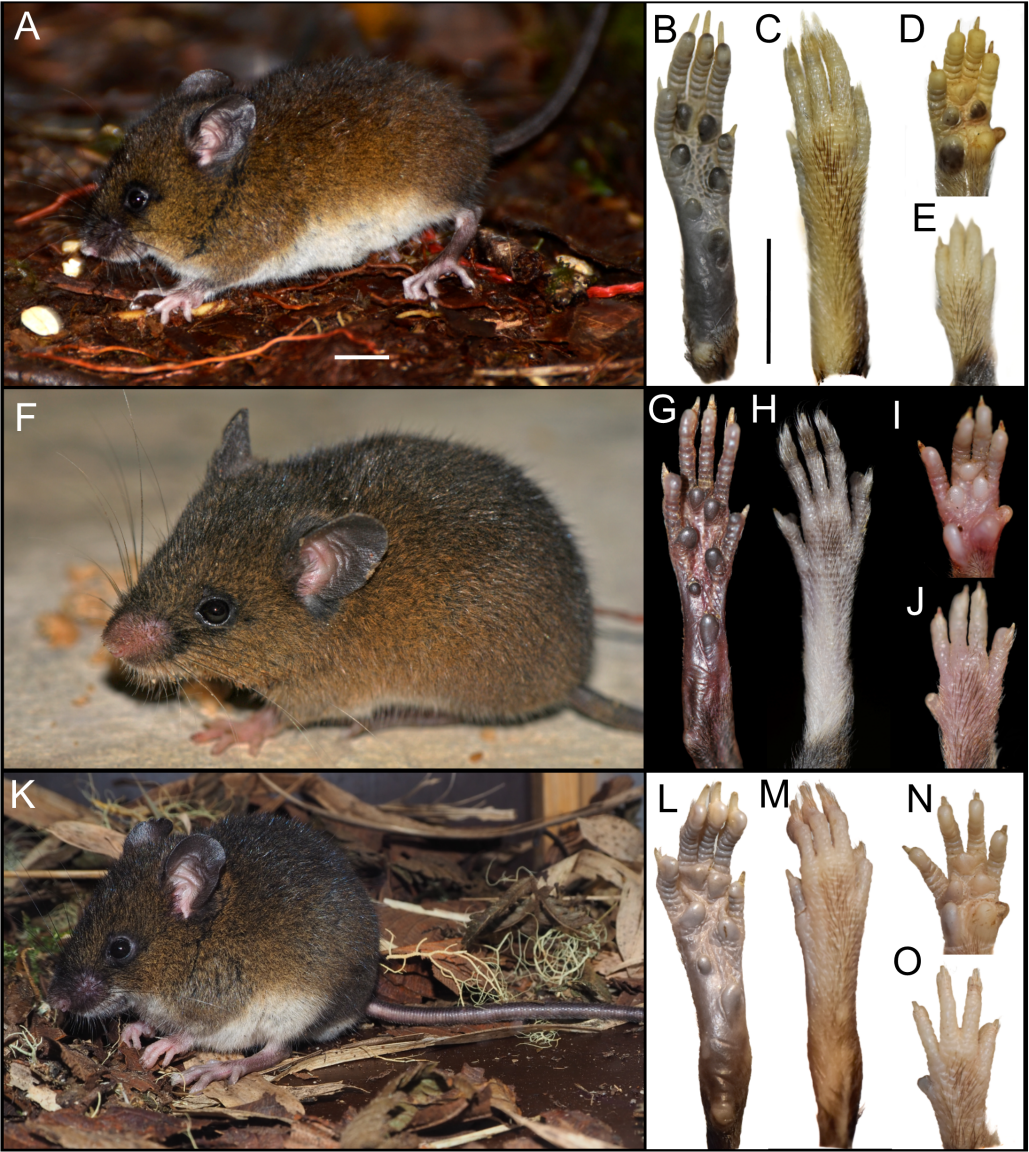
External appearance of the three *Oreoryzomys* species (left panels) and details of their feet and hands (right panels). Top row: (A) live lateral view (*O. balneator*, MECN 5815); (B–C) plantar and dorsal views of foot; (D–E) palmar and dorsal views of hand (MECN 6140). Middle row: (F) live lateral view (*O. a. hesperus*, MECN 4789); (G–H) plantar and dorsal views of foot; (I–J) palmar and dorsal views of hand. Bottom row: (K) live lateral view (*O.* sp. nov, MECN 8278, holotype); (L–M) plantar and dorsal views of foot; (N–O) palmar and dorsal views of hand. Scale = 10 mm. Photographs (A–J, L–O) by J Brito; (K) by R Wistuba.

The newly collected material provides information on several aspects of external and soft anatomy, as well as postcranial skeletal features. *Oreoryzomys* exhibits a relatively simple, bean-shaped pinna, which is easily visible above the fur on the head and possesses a well-developed concha (Figs. 3A, 3F, 3K). The inner surface of the pinna is nearly hairless and unpigmented, with the margins sparsely covered by fine, dark hairs.

**Figure 4:**
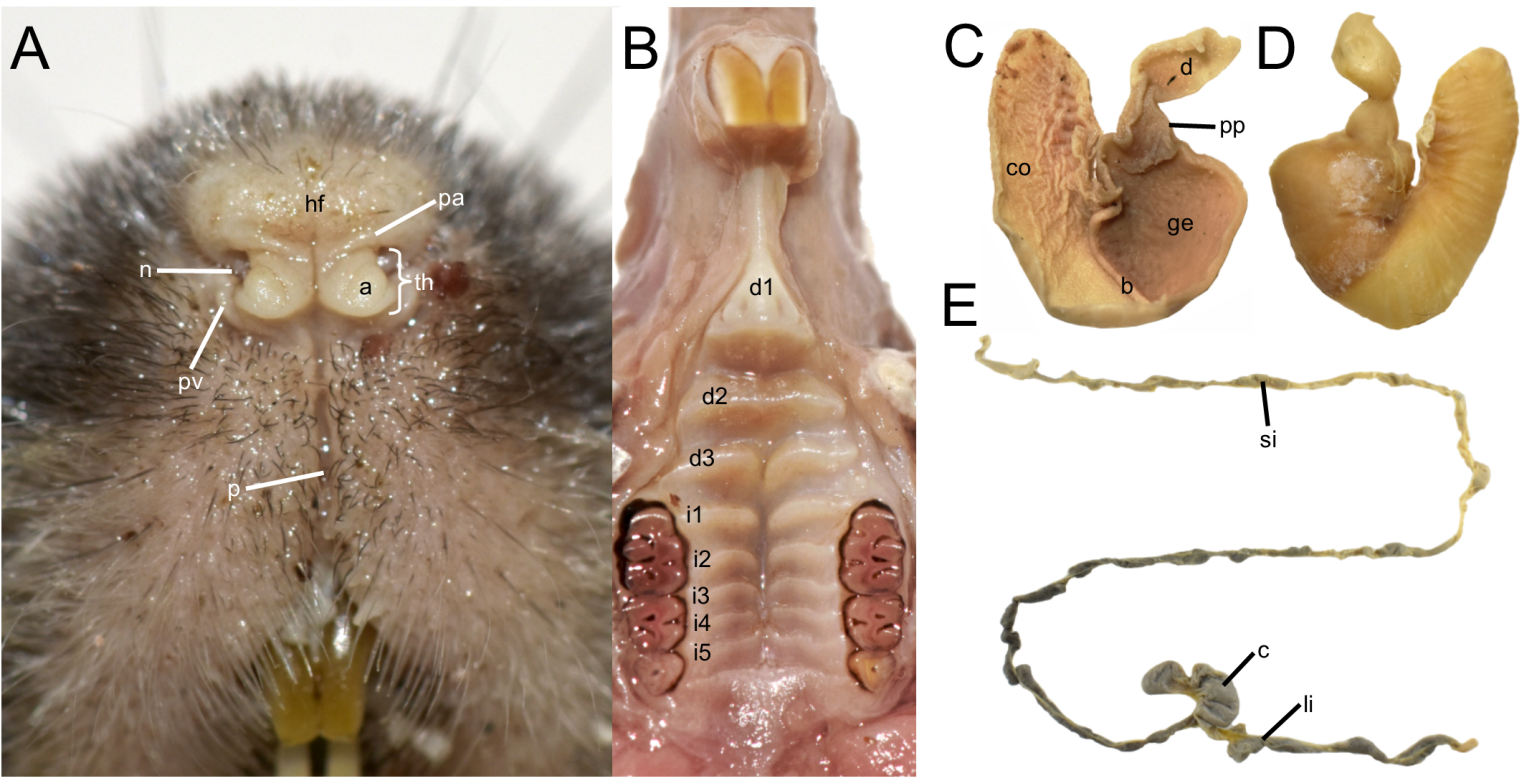
Selected external and soft anatomical features of *Oreoryzomys balneator* (MECN 7346). (A) Rhinarium and upper lip in ventral view; (B) soft palate; (C–D) stomach in internal and external views, respectively; (E) external view of partial digestive tract. Abbreviations: a, areola circularis; b, bordering fold; c, cecum; co, cornified epithelium; d, duodenum; d1–d3, diastemal rugae; ge, glandular epithelium; hf, haired fold; i1–i5, interdental rugae; li, large intestine; n, nostril; p, philtrum; pp, pyloric part; pa, plica alaris; pv, plica ventralis; si, small intestine; th, Tubercle of Hill. Photographs by J Brito.

The rhinarium bears small lateral nostrils and is dorsally overlain by a slight tuft of hairs. Its overall configuration corresponds to the “grapeseed” type, a form typically observed in oryzomyine rodents. The haired fold is well developed and appears largely naked. The areola circularis is small and crossed by faint transverse grooves that define the crus superius and crus inferius, collectively forming a distinct tubercle of Hill. The plica ventralis is also well developed (Fig. 4A).

The soft palate exhibits three diastemal folds, with the first well-protruding and triangular in outline and the third anteriorly indented by a medial cleft although not divided. The palatine (or interdental) rugae number at least five, medially divided and display moderate relief (Fig. 4B).

The anatomical study confirms the absence of a gallbladder in *Oreoryzomys*. The stomach corresponds to the unilocular-hemiglandular type (Figs. 4C–4D). A prominent bordering fold divides the stomach into two roughly equal halves. The glandular epithelium extends only slightly beyond the esophageal orifice on the left side. The corpus is almost entirely lined with coarse, cornified epithelium, and the pyloric part is well developed. The small intestine measures approximately 350 mm, and the large intestine about 80 mm in length (Fig. 4E). The cecum appears as a single pouch of approximately 30 mm length; this organ is composed of a bulbous ampulla coli and the body (corpus ceci) lacking any indication of haustra-like bulges and appendix vermiformis (Fig. 4C).

**Figure 5:**
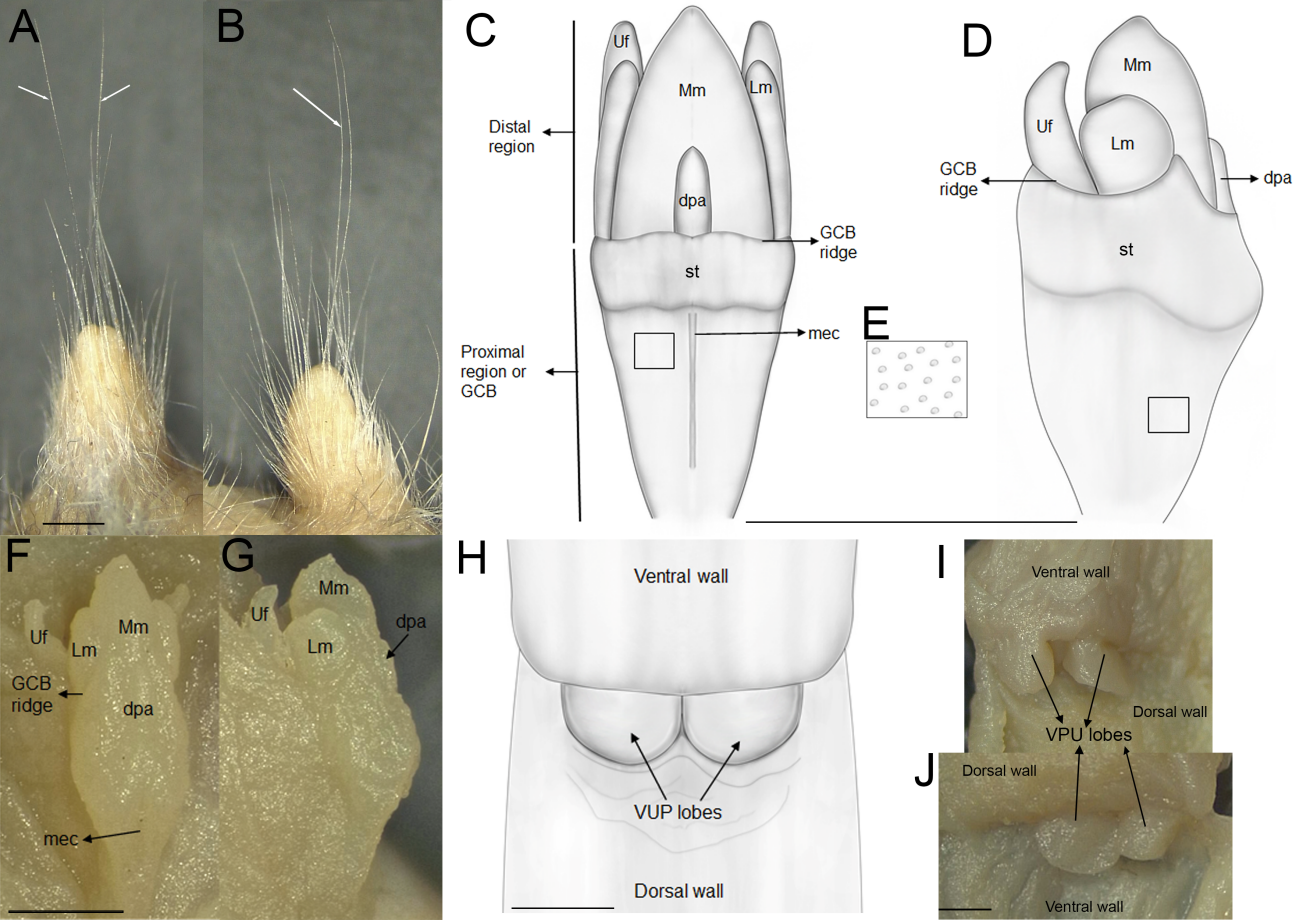
External and internal genital anatomy of female *Oreoryzomys balneator* (MECN 6140, province Tungurahua, Ecuador). (A) Dorsal view of the external prepuce; (B) lateral view of the external prepuce; (C) dorsal schematic view of the glans clitoris; (D) lateral schematic view of the glans clitoris; (E) tubercles of the GCB; (F) dorsal view of the clitoris; (G) lateral view of the clitoris; (H) ventral schematic view of the vaginal-uterine portion; (I) ventral view of the vaginal-uterine portion; (J) dorsal view of the vaginal-uterine portion. Abbreviations: dpa, dorsal papilla; GCB, glans clitoris body; Lm, lateral mound; mec, medial cleft; Mm, medial mound; st, soft tissue; VUP, vaginal-uterine portion; Uf, urethral flaps. White arrows indicate cerci. Scale = one mm. Orientation: upper margin = anterior, left margin = ventral. Photographs by C Nivelo-Villavicencio.

The external female genital system is characterized by a short prepuce, slightly longer (2.5 mm) than wide (2.2 mm), with an acuminate distal portion forming an elongated “U” shape. The dorsal surface is sparsely covered with whitish, translucent hairs (Figs. 5A–5B), some with brownish bases, extending laterally onto the flanks but absent along the preputial meatus margin. The ventral surface mirrors this pattern, lacking a defined raphe and presenting only a shallow basal depression. At the distal end of the external prepuce lies the posteriorly positioned preputial meatus, with smooth, glabrous margins. It opens dorsally and ventrally, producing a bifid appearance. Adjacent to the dorsal margin are two pores, each giving rise to a cercus that exceeds the prepuce in length and shares similar coloration with the preputial hairs. Internally, the glans clitoris, approximately 1.3 mm long and 0.35 mm wide, consists of a proximal glans body and a distal mound region, separated by a ridge. The medial mound, the most prominent structure (0.58 mm), is acuminate, with a broad base and a tapering distal tip. Two less prominent lateral mounds flank the medial mound. In dorsal view, they are longer than wide, whereas in lateral view they appear rounded and partially overlap the medial mound. A lanceolate dorsal papilla is situated at the base of the medial mound. Ventral to the mounds lie the urethral flaps, resembling the lateral mounds in dorsal view and presenting narrow, concave, cone-like profiles in lateral view (Figs. 5F–5G). Anterior to these structures is the glans clitoris body, which exhibits an irregular dorsal contour with globular elevations and a deep medial cleft dividing it into two lateral bodies (Figs. 5C–5D). These bear sparse dorsal tubercles, absent from the cleft and distal third, where a band of smooth, non-spinous tissue is present. The glans body fuses with the inner surface of the prepuce along its margin. The vagina is a dorsoventrally concave canal (∼7.0 mm long, 3.5 mm wide). At the top portion lies the cervicovaginal junction. In this region, two smooth and symmetrical U-shaped lobes are present, visible in both ventral and dorsal views.

No previous data on the postcranial skeleton of *Oreoryzomys* are available. In the specimens studied (*n* = 14), the tubercle of the first rib articulates with the transverse processes of both the seventh cervical and the first thoracic vertebrae. The second thoracic vertebra possesses a distinctly elongated neural spine. The vertebral formula includes 19 thoracolumbar, four sacral, and 29–36 caudal vertebrae, with complete hemal arches present on the second and third caudal vertebrae. The rib count is 12.

In accordance with the genetic, morphometric, and morphological results, we consider that the genus *Oreoryzomys* comprises three species, which are presented below.

### Systematics

**Table:** 

Family Cricetidae Fischer, 1817
Subfamily Sigmodontinae Wagner, 1843
Tribe Oryzomyini Vorontsov, 1959
Genus *Oreoryzomys*Weksler, Percequillo & Voss, 2006

### *Oreoryzomys balneator* (Thomas, 1900)

*Oryzomys balneator* Thomas 1900: 273. Type locality: “Mirador, 20 miles E. of Baños, Oriente of Ecuador. Altitude 1,500 m” (Thomas, 1900: 274).

**Figure 6:**
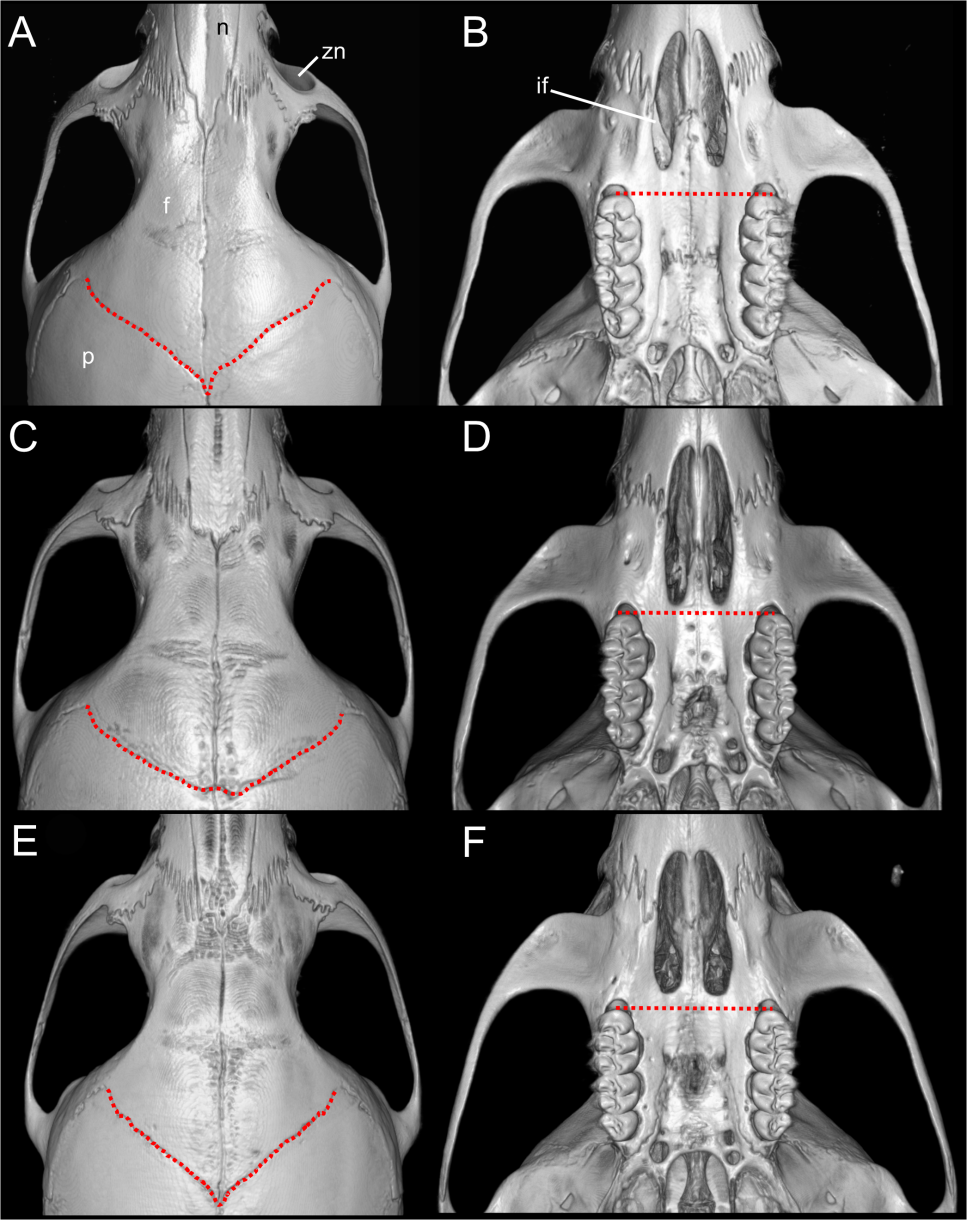
Selected qualitative anatomical features in the crania of *Oreoryzomys balneator* (A–B) MECN 5795), *O. hesperus* (C–D) MECN 4789), and *O. jumandi* sp. nov. (E–F) MECN 8278, holotype), scaled to the same length. Coronal sutures and the plane defined by the anterior faces of the first upper molars are highlighted in red. Abbreviations: n, nasal; if, incisive foramen; f, frontal; p, parietal; zn, zygomatic notch. 3D reconstruction by C Koch and J Brito.

**Emended diagnosis**: A species of *Oreoryzomys* distinguished by the following combination of characters: incisive foramina markedly short, not extending to the anterior margin of M1 (Fig. 6B); frontoparietal (coronal) suture distinctly V-shaped (Fig. 6A); stapedial process of the auditory bulla extremely reduced or nearly imperceptible; median lacerate foramen narrow and partially occluded by the posterior extension of the pterygoid plate; auditory bulla in direct contact with the alisphenoid; M3 with hypoflexus reduced to a shallow notch; and m2 with a short mesolophid that does not reach the mesostylid.

**Figure 7:**
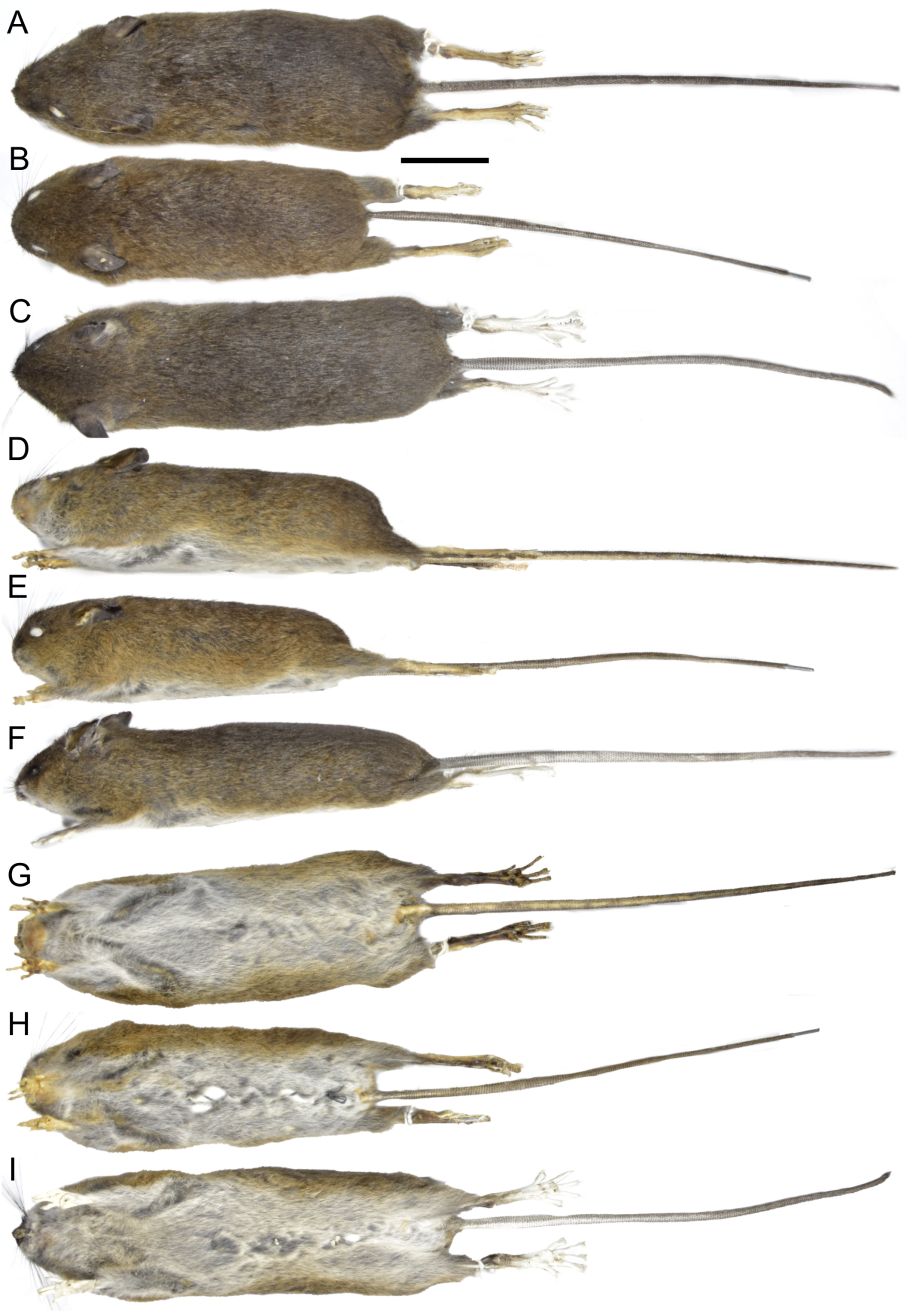
External appearance based on museum skins in dorsal (A–C), ventral (D–F), and lateral (G–I) views. (A, D, G) *Oreoryzomys balneator* (MECN 5801; Cordillera de Kutukú, Ecuador); (B, E, H) *Oreoryzomys hesperus* (MECN 4789; Cordillera de Chilla, Ecuador); (C, F, I) *Oreoryzomys jumandi* sp. nov. (MECN 8278; Estación Científica Yanayacu, Napo, Ecuador). Scale = 25 mm. Photographs by J Brito.

**Description:** Dorsal pelage dark brown, with individual hairs measuring 8–9 mm in length, basally gray and apically orange. Ventral fur sharply contrasting with the dorsum (Fig. 7A), composed of shorter hairs (4–5 mm), basally gray and apically white. Ears comparatively short, measuring 12–19 mm. Tail moderately short, ranging from 108–131 mm in length (averaging 118% of head-and-body length), either unicolored (entirely dark) or indistinctly bicolored at the base.

**Figure 8:**
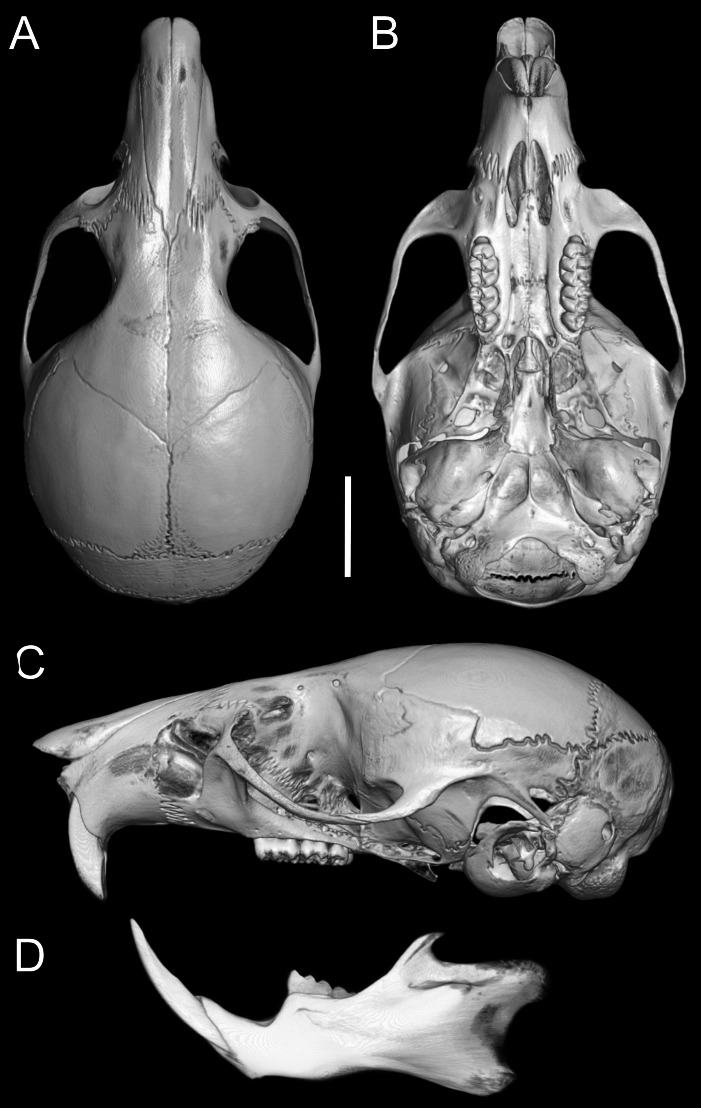
Cranium and mandible. Cranium in dorsal (A), ventral (B), and lateral (C) views, and mandible in labial view (D) of *Oreoryzomys balneator* (MECN 5795; Reserva Vizcaya, Tungurahua, Ecuador). Scale = five mm. 3D reconstruction by C Koch and J Brito.

**Figure 9:**
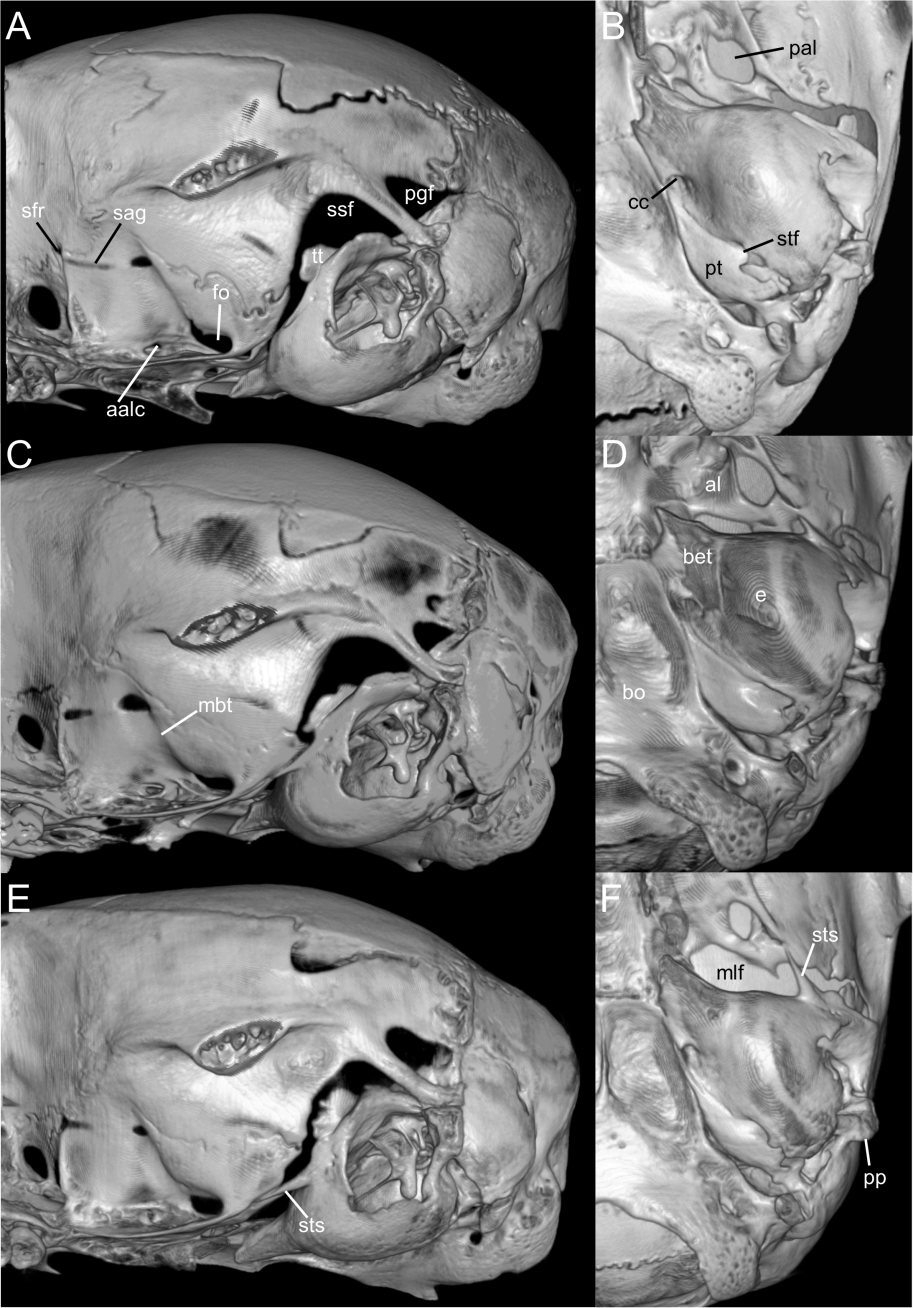
Selected qualitative anatomical features in the crania of *Oreoryzomys balneator* (A–B) MECN 5795), *O. hesperus* (C–D) MECN 4789), and *O. jumandi* sp. nov. (E–F) MECN 8278, holotype), scaled to the same length. Coronal sutures and the plane defined by the anterior faces of the first upper molars are highlighted in red. Abbreviations: n, nasal; if, incisive foramen; f, frontal; p, parietal; zn, zygomatic notch. 3D reconstruction by C Koch and J Brito.

Skull (Figs. 6A–6B, 8, 9A–9B) with rounded cranial profile, especially along braincase; temporal ridges absent (Figs. 8A, 8C). Nasals extend posteriorly beyond plane of lacrimals. Zygomatic notch deep. Fronto-parietal (coronal) suture V-shaped (Fig. 6A). Alisphenoid strut absent (Fig. 9A); buccinator–masticatory foramen confluent with accessory foramen ovale. Stapedial foramen, squamosal–alisphenoid groove, and sphenofrontal foramen present (carotid pattern 1; Voss, 1988). Incisive foramina broad, short, with thick medial septum; posterior margin anterior to M1 (Fig. 6B). Middle lacerate foramen narrow, partially closed by parapterygoid plate and auditory bulla. Auditory bulla small, slightly inflated; stapedial process minute, not reaching alisphenoid (Table 4).

**Figure 10:**
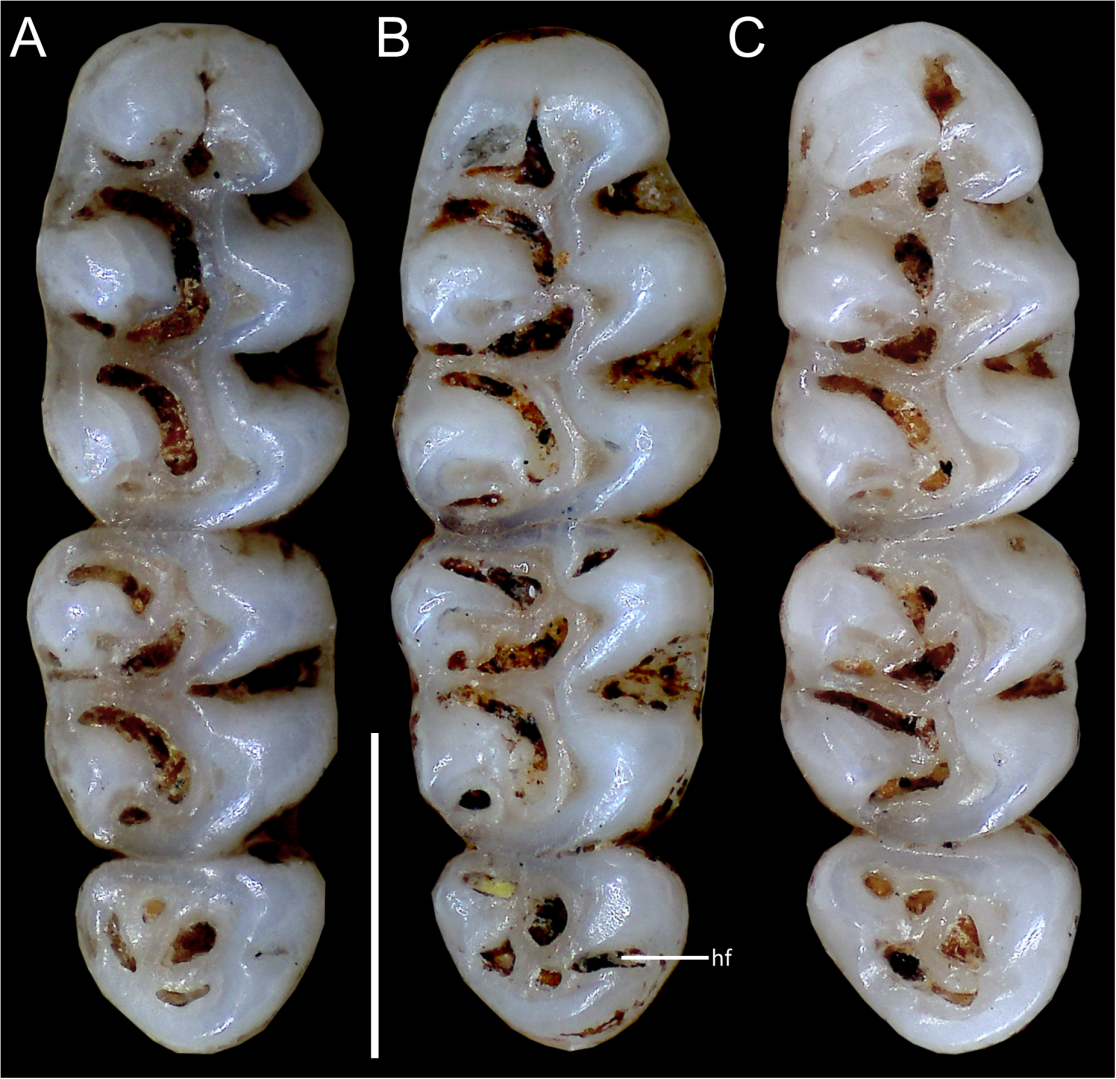
Comparative morphology of upper right molar series in occlusal view among *Oreoryzomys* species: (A) *O. balneator* (MECN 5009); (B) *O. hesperus* (MECN 4789); (C) *O. jumandi* sp. nov. (MECN 8278, holotype). Abbreviation: hf, hypoflexus. Scale = one mm. Photographs by J Brito.

**Figure 11:**
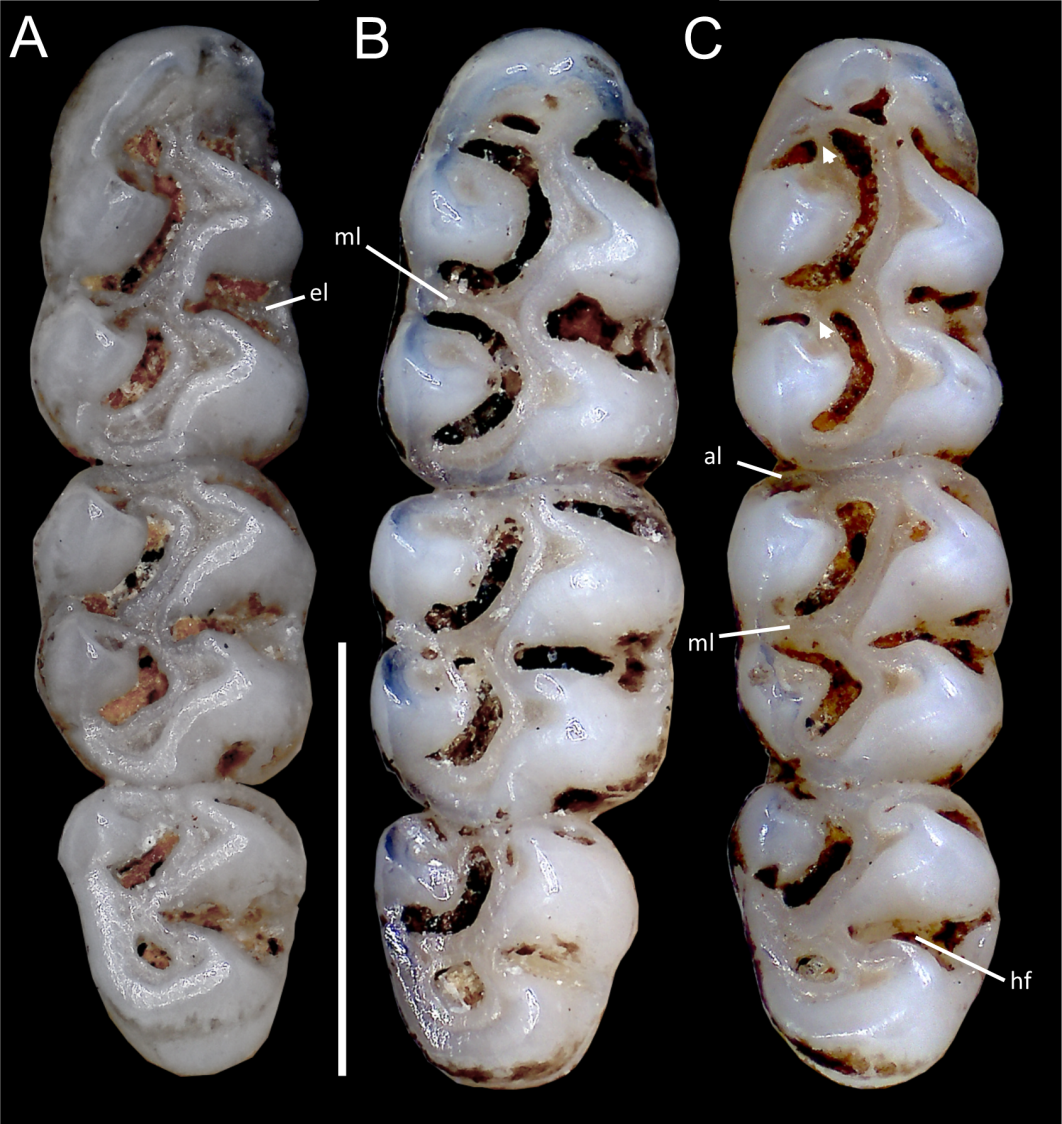
Comparative morphology of lower right molar series in occlusal view among *Oreoryzomys* species: (A) *O. balneator* (MECN 5009); (B) *O. hesperus* (MECN 4789); (C) *O. jumandi* sp. nov. (MECN 8278, holotype). The arrows indicate the connection between the metaconid and the anterolophid, and the connection between the entoconid and the mesolophid. Abbreviations: al, anterolophid; el, ectolophid; hf, hypoflexid; ml, mesolophid. Scale = one mm. Photographs by J Brito.

Upper incisors with orange-colored enamel. M1 with anteromedian flexus dividing procingulum into two conules; labial conule smaller than lingual; paracone and protocone isolated from median mure by large, recurved flexi; M2 with hypoflexus and metaflexus, both reaching midline; M3 with very shallow hypoflexus, appearing as minor indentation (Fig. 10A); m1 with poorly developed anterolophid; mesolophid small, not contacting mesostylid; m2 with small mesolophid; m3 with long, deeply infolded hypoflexid (Fig. 11A).

**Distribution:** The known distribution of *Oreoryzomys balneator* extends from central-southeastern to southwestern Ecuador, with confirmed records from the provinces of Morona Santiago and Tungurahua. Specimens have been collected at elevations ranging from 1,500 to 2,820 m above sea level.

**Natural history:** *Oreoryzomys balneator* inhabits the Eastern Subtropical and Temperate zoogeographic regions of Ecuador (Albuja et al., 2012). Its habitat corresponds to montane forest ecosystems (Ministerio del Ambiente del Ecuador, 2013), characterized by a dense canopy of trees often covered with epiphytic orchids, ferns, and bromeliads. Specimens have been collected in mature forest, where the understory is visually dominated by herbaceous plants belonging to families such as Poaceae (notably *Chusquea* spp.), Araceae, and Melastomataceae. On steep slopes, the royal palm (*Dictyocaryum lamarckianum*) is often the dominant tree species.

*Oreoryzomys balneator* occurs in syntopy with a diverse assemblage of small mammals, including the didelphids *Marmosa perplexa*, *Marmosops caucae*, and *Monodelphis adusta*, as well as several cricetid rodents: *Akodon aerosus*, *A. mollis*, *Mindomys kutuku*, *Nephelomys auriventer*, *N. nimbosus*, *Rhipidomys albujai*, *Thomasomys pardignasi*, *T. cinnameus*, *T. erro*, and *T. salazari*.

**Specimens examined** **(n = 72): Ecuador, Morona Santiago**, 9 de Octubre (MECN 5651, 5687), Cordillera de Kutukú (MECN 5795, 5801–02, 5815, 5837–47, 5860, 5862, QCAZ 17572, 17576, 17579, 17573), Sardinayacu (MECN 3800, 3802); Tinguichaca (QCAZ 20342–3, 20345); **Tungurahua**, Cerro Mayordomo (MECN 8016, 8018, 8020), Cerro Candelaria (MECN 5008–9, 5012–13, 5015–17, 5019–21, 5023–24), Guamag (MECN 7219–20, 7226, 7228, 7230, 7235, 7237, 7242, 7252, 7254–55), La Palmera (MECN 7187, 7189, 7192, 7194, 7198, 7217), Machay (MECN 1836), Chamana Pamba (MECN 6469, 6487, 7346), Reserva Vizcaya (MECN 6140, 6343, 6345–46, 6404, 6414).

**Table 4:** Morphological comparisons of selected traits among species of the genus of rodent *Oreoryzomys*.

**Character**	***O. balneator***	***O. hesperus***	***O. jumandi*** ** sp. nov.**
Back hair	8–9 mm	6–7 mm	7–8 mm
Frontoparietal suture	V-shaped	U-shaped	V-shaped
Incisive foramen	Short, without reaching the base of the M1	Long, reaches the root of M1	Short, without reaching the root of M1
Stapedial process of bulla	Short, without reaching the edge of the alisphesnoid	Medium, just reaches the edge of the alisphesnoids	Long, it goes beyond the edge of the alisphesnoid
Middle lacerate foramen	Narrow, bulla in contact with alisphenoid	Narrow, bulla in contact with alisphenoid	Wide, distant bulla of the alisphenoid
Tegmen timpanic	Short and narrow	Short and wide	Short and wide
Hypoflexus of M3	Shallow	Deep, reaching the central fossette	Shallow
Mesolophid of m1	Small, fused to the entoconid	Long, fused to the median mure	Long, fused to the median mure and the entoconid
Mesolophid of m2	Small, fused to entoconid and median mure	Small, fused to entoconid and median mure	Long, fused to the median mure
Hypoflexid of m3	Long, reaching to the mesophlexid and posteroflexid	Short, without reaching mesophlexid and posteroflexid	Long, reaching to the mesophlexid and posteroflexid

### *Oreoryzomys hesperus* (Anthony, 1924) nov. comb.

*Oryzomys balneator hesperus* Anthony, 1924: 7. Type locality: “El Chiral, Western Andes, Provincia del Oro, Ecuador, elevation 5350 ft” (Anthony, 1924: 7).

**Emended diagnosis:** A species of *Oreoryzomys* characterized by the following combination of traits: incisive foramina elongated, reaching the anterior root of M1 (Fig. 6D); frontoparietal (coronal) suture broadly U-shaped (Fig. 6C); stapedial process of the auditory bulla moderately developed, extending anteriorly to contact the alisphenoid; median lacerate foramen narrow; auditory bulla in direct contact with the alisphenoid; M3 with a well-developed, penetrant hypoflexus (Fig. 10B); and m2 bearing a small mesolophid that contacts both the mesostyle and the entoconid (Fig. 11B).

**Description:** Dorsal pelage dark brown (Fig. 7B); hairs 6–7 mm, basally gray, apically orange. Ventral fur contrasting; hairs 4–5 mm, basally gray, apically white. Ears rounded, 15–18 mm, covered externally with short blackish hairs. Tail 110–124 mm (averages 117% of head-body length), unicolored, blackish. Caudal scales rectangular, each with two hairs extending over 1.5–2 rows. Mystacial vibrissae ∼31 mm, slender, reaching beyond pinnae; genal vibrissae present.

Manus with five digits: digit I with reduced, broad claw; digits II–V with long, curved claws. Palmar surface with five pads: thenar rounded and broad; hypothenar elongated; interdigital pads small, rounded (Fig. 3I). Hind feet 24–28 mm, mostly whitish with faint dark patch on distal metatarsals. Ungual tufts well developed, exceeding claws. Plantar surface with six pads: four interdigital pads of similar size; hypothenar ∼$ \frac{1}{4} $ size of thenar. Surface between pads granular (Fig. 3G).

**Figure 12:**
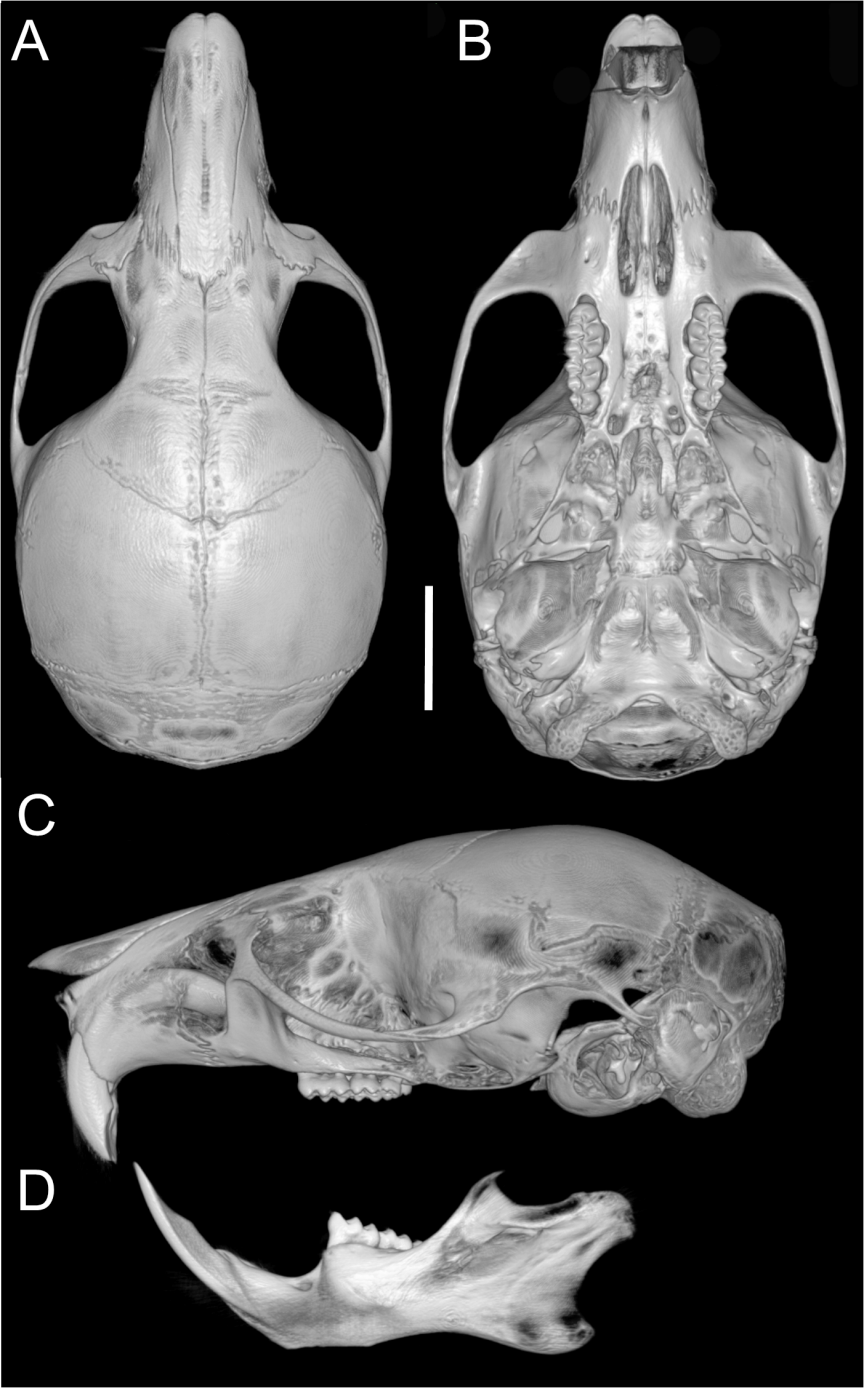
Cranium and mandible. Cranium in dorsal (A), ventral (B), and lateral (C) views, and mandible in labial view (D) of *Oreoryzomys hesperus* (MECN 4789; Cordillera de Chilla, El Oro, Ecuador). Scale = five mm. 3D reconstruction by C Koch and J Brito.

Skull (Figs. 6C–6D, 9C–9D, 12) small (CIL 19.84–23.18 mm); cranial profile rounded; temporal ridges absent. Rostrum moderately broad (BR/ZB = 36% ± 1.4), slightly elongated. Nasals extend beyond lacrimals; zygomatic notch shallow (Fig. 6C). Interorbital region wide; frontoparietal suture U-shaped (Fig. 6C). Braincase wide, rounded (Fig. 12A); interparietal broad. In lateral view, nasals extend beyond incisors; gnatic process reduced. Posterior edge of zygomatic plate aligned with M1 root. Postglenoid foramen small relative to subsquamosal fenestra (Fig. 9C). Hamular process of squamosal thin, overlapping mastoid capsule distally. Alisphenoid strut absent; buccinator–masticatory foramen confluent with accessory foramen ovale. Stapedial foramen, squamosal–alisphenoid groove, and sphenofrontal foramen present (carotid pattern 1; Voss, 1988). Incisive foramina large, extending posteriorly to but not between M1 alveoli (Fig. 6D); widest posteriorly; lateral margins parallel. Posterolateral palatal pits large, recessed in shallow fossae (Fig. 12B). Mesopterygoid fossa extends anteriorly between maxillae, not between molar rows; bony roof with short sphenopalatine vacuities. Middle lacerate foramen narrow; auditory bulla in contact with alisphenoid. Auditory bullae small, slightly inflated; stapedial process medium-sized, just reaches alisphenoid edge. Capsular process of lower incisor located below coronoid base. Superior and inferior masseteric ridges converge anteriorly below m1. Angular process short, not reaching condylar process; angular notch shallow (Fig. 12D).

Upper incisors with orange enamel. M1 with anteromedian flexus dividing procingulum into two conules; anterolabial smaller than anterolingual; anteroloph present, separate from anterolabial conule; paracone and protocone joined by enamel bridge; mesoloph long, narrow, connected to metacone *via* thin enamel bridge; M2 with protoflexus; hypoflexus and metaflexus not interpenetrated; M3 lacks posteroloph; hypoflexus deep, reaching central fossette (Fig. 10B); M1 with three roots (no accessory labial root); M2 and M3 with two roots; m1 with shallow anteromedian flexid on anteroconid; distinct anterolophid; long mesolophid; entoconid not connected to median mure; m2 lacks anterolophid; small mesolophid partially fused to entoconid (Fig. 11B); m3 small, slightly square; anterolophid poorly developed; lower molars 2-rooted.

**Distribution:** Examined specimens of *Oreoryzomys hesperus* document a geographic range that extends from south-central and southwestern Ecuador (provinces of Azuay, El Oro, and Zamora Chinchipe) to northwestern Peru (Cajamarca). Recorded elevations range from 1,500 to 2,630 m. Although no Peruvian specimens were directly examined, two sequences available in GenBank (https://www.ncbi.nlm.nih.gov/nuccore/?term=Oreoryzomys%2C+Peru) are phylogenetically resolved as closely related to *O. hesperus* and are thus considered representative of the species in this study.

**Natural history:** *Oreoryzomys hesperus* inhabits the Temperate zoogeographic region as defined by Albuja et al. (2012). The species occurs in montane forest ecosystems (Ministerio del Ambiente del Ecuador, 2013), which are characterized by dense tree cover with abundant epiphytes, including orchids, ferns, and bromeliads. Specimens were collected in mature forest, where the understory is visually dominated by herbaceous families such as Poaceae (notably *Chusquea* spp.), Araceae, and Melastomataceae. On steep slopes, palms *Ceroxylon* are dominant.

*Oreoryzomys hesperus* was found in sympatry with several small mammals, including the marsupials *Marmosops caucae*, *Caenolestes caniventer*, and *C. condorensis*, and the cricetid rodents *Akodon mollis*, *Nephelomys albigularis*, *Microryzomys minutus*, and *Thomasomys taczanowskii*.

**Remarks:** Although no information is given on the origin of the name of the subspecies he redescribed, Anthony (1924) possibly used *hesperus* as a toponymic reference to the species’ occurrence west of the Andes. Hesperus was associated with the evening star (Venus) and, in Roman usage, also with the west, owing to the setting sun.

**Specimens examined (*n* = 27):** **Ecuador, Azuay**, Amaluza (MECN 7932); **El Oro**, Chivaturco (MECN 4789); **Zamora Chinchipe**, Los Rubíes (MECN 2479), Numbala Alto (MECN 1341, 1344), Loma Chumasquín (QCAZ 13228), Reserva Biológica Tapichalaca (MECN 3724, QCAZ 18990–95, 18997–98, 19000, 19003–04, 19006–07), Sabanilla (QCAZ 13178–82), Yacuambi (QCAZ 13280), El Denuncio (QCAZ 13196).

**Table:** 

***Oreoryzomysjumandi*** new species. Brito, Vargas, García, Tinoco & Pardiñas
*Oreoryzomys balneator*: Lee Jr et al., 2006, not *balneator* (Thomas, 1900).
urn:lsid:zoobank.org:act:86B4AE13-FF63-437E-89E8-516B1BE96F0F
Jumandi Mountain Mouse, Ratón montano de Jumandi (in Spanish)

**Holotype:** MECN 8278, an adult male collected on 14 October 2024 by R. Wistuba. The specimen is preserved as a dry skin, skull, postcranial skeleton, and muscle and liver tissue samples stored in 95% ethanol.

**Type locality:** Ecuador, Provincia de Napo, Cantón Quijos, Estación Biológica Yanayacu (−0.599496°, −77.890374°, WGS84 coordinates taken by GPS at the site of collection; elevation 2,220 m).

**Etymology**: Named in honor of Jumandi, a Quijo warrior who led the first indigenous uprising against Spanish conquistadors in the Americas on 29 November 1578 (Santos-Granero, 1992). In recognition of his historical significance, Jumandi was officially declared a National Hero by the Asamblea Nacional del Ecuador in November 2011.

**Diagnosis:** A species of *Oreoryzomys* distinguished by the following combination of characters: incisive foramina short, not reaching the anterior margin of M1 (Fig. 6F); frontoparietal (coronal) suture distinctly V-shaped (Fig. 6E); stapedial process of the auditory bulla elongate and pointed, projecting beyond the posterior margin of the alisphenoid (Fig. 9F); median lacerate foramen broad and positioned at a distance from the bulla; M3 with the hypoflexus shallow, forming a lake-like structure; and m2 with a long mesolophid fused to the mesostyle (Fig. 11C).

**Morphological description of the holotype and variation:** Dorsal coat dark brown (Fig. 7C); hairs 7–8 mm long, basally gray, apically whitish. Ventral fur clearly distinct, hairs 4–5 mm, basally gray, apically whitish. Ears 16–19 mm (Table 5), rounded, covered externally with short blackish hairs. Tail 110–124 mm (138% head-body length in average), blackish, unicolored, slightly bicolored base; caudal scales rectangular, three hairs each, extending 1–1.5 rows. Mystacial vibrissae ∼35 mm, slender, reaching beyond pinna when tilted back. Genal vibrissae present.

**Table 5:** Univariate statistics (minimum, maximum and mean standard deviation) of external and craniodental measurements (in mm), and weight in grams for each species of the genus *Oreoryzomys*. Acronyms of variables are explained in the main text (Materials and Methods section).

	***O. balneato*** **r (*n* = 25)**	***O. hesperus*** **(*n* = 21)**	***O. jumandi*** **sp. nov. (*n* = 32)**	
			**Holotype MECN 8278**	**Paratypes**
HBL	62–125 (84 ± 13.79)	81–93 (85 ± 3.57)	88	73–94 (84.42 ± 5.73)
TL	108–131 (115.47 ± 9.79)	110–122 (117.56 ± 4.39)	115	110–124 (116.21 ± 5.07)
HF	19–27 (24.27 ± 2.31)	24–29 (26.44 ± 1.81)	25	25–31 (27.04 ± 1.46)
E	12–19 (15.53 ± 1.82)	15–18 (16.67 ± 0.87)	17	16–19 (17.54 ± 1.03)
W	32–39 (35.67 ± 2.34)	19–33 (24.56 ± 5.13)	27	22–40 (30.58 ± 4.13)
ONL	21.36–25.9 (23.9 ± 1)	21.92–26.21 (24.65 ± 1.02)	23.93	22.67–26.08 (24.65 ± 0.84)
CIL	19.82–23.53 (22.08 ± 0.82)	19.84–23.18 (21.9 ± 0.86)	22.24	21.02–23.49 (22.31 ± 0.67)
LD	5.47–7.06 (6.32 ± 0.38)	5.58–6.54 (6.07 ± 0.29)	6.62	5.48–6.69 (6.16 ± 0.32)
LM	2.96–3.59 (3.31 ± 0.17)	3.1–3.45 (3.28 ± 0.09)	3.27	3.15–3.42 (3.26 ± 0.07)
LIF	3.16–4.2 (3.55 ± 0.24)	3.1–3.95 (3.46 ± 0.23)	3.50	2.98–3.75 (3.34 ± 0.19)
BIF	1.54–2 (1.81 ± 0.12)	1.35–1.98 (1.62 ± 0.17)	1.73	1.63–1.97 (1.76 ± 0.1)
LN	8.1–10.41 (9.41 ± 0.65)	7.93–9.87 (8.91 ± 0.6)	9.36	8.65–9.79 (9.25 ± 0.3)
LPB	3.81–5.57 (4.4 ± 0.33)	3.96–4.67 (4.36 ± 0.21)	4.03	3.64–4.36 (4.06 ± 0.18)
BBP	2.1–3.04 (2.47 ± 0.22)	2.22–2.73 (2.46 ± 0.13)	2.76	1.5–2.76 (2.11 ± 0.29)
LIB	4.29–4.96 (4.56 ± 0.15)	4.06–4.6 (4.38 ± 0.14)	4.73	4.25–4.95 (4.58 ± 0.14)
ZB	11.59–14.08 (12.75 ± 0.49)	11.55–13.46 (12.7 ± 0.45)	13.32	12.52–14.16 (13.39 ± 0.41)
BZP	1.69–2.23 (1.95 ± 0.15)	1.75–2.1 (1.94 ± 0.11)	2.13	1.78–2.9 (2.1 ± 0.2)
OFL	6.52–7.86 (7.17 ± 0.31)	6.54–7.56 (7.09 ± 0.28)	7.68	7.07–8.02 (7.54 ± 0.24)
BL	2.3–4.42 (3.75 ± 0.66)	2.21–4.18 (2.76 ± 0.49)	4.36	2.09–4.36 (2.62 ± 0.62)
LJ	11.43–13.46 (12.57 ± 0.57)	10.96–13.16 (12.11 ± 0.52)	12.2	11.39–13.18 (12.1 ± 0.34)
LMI	3.22–3.71 (3.49 ± 0.15)	3.19–3.68 (3.45 ± 0.11)	3.51	3.22–3.57 (3.43 ± 0.09)
LDI	2.58–3.54 (3.13 ± 0.25)	2.31–3.39 (2.82 ± 0.23)	3.34	2.08–3.61 (2.65 ± 0.37)
LM1	1.25–1.63 (1.5 ± 0.1)	1.45–1.79 (1.57 ± 0.09)	1.50	1.39–1.69 (1.55 ± 0.08)
WM1	0.8–1.14 (1.01 ± 0.08)	0.89–1.11 (1 ± 0.05)	1.06	0.91–1.06 (1 ± 0.04)
LM2	0.72–1.06 (0.89 ± 0.08)	0.88–1.12 (1.01 ± 0.07)	0.77	0.77–1.12 (0.98 ± 0.07)
WM2	0.85–1.11 (0.98 ± 0.07)	0.83–1.04 (0.92 ± 0.07)	0.99	0.86–1.01 (0.95 ± 0.04)
LM3	0.53–0.76 (0.65 ± 0.06)	0.55–0.79 (0.66 ± 0.06)	0.65	0.63–0.79 (0.7 ± 0.04)
WM3	0.53–0.92 (0.78 ± 0.09)	0.67–0.85 (0.76 ± 0.05)	0.85	0.77–0.91 (0.84 ± 0.03)
Lm1	1.22–1.56 (1.38 ± 0.09)	1.33–1.56 (1.45 ± 0.06)	1.36	1.32–1.51 (1.42 ± 0.05)
Wm1	0.7–1.08 (0.95 ± 0.08)	0.78–0.94 (0.87 ± 0.05)	0.99	0.87–0.99 (0.94 ± 0.03)
Lm2	0.8–1.22 (1.02 ± 0.13)	0.95–1.16 (1.08 ± 0.05)	0.89	0.89–1.11 (1.03 ± 0.04)
Wm2	0.78–1.05 (0.94 ± 0.08)	0.87–1 (0.92 ± 0.03)	0.98	0.83–0.99 (0.94 ± 0.03)
Lm3	0.77–1 (0.88 ± 0.06)	0.75–1.01 (0.89 ± 0.06)	1.00	0.85–1.04 (0.96 ± 0.04)
Wm3	0.65–0.85 (0.78 ± 0.06)	0.61–0.83 (0.75 ± 0.05)	0.83	0.78–0.92 (0.84 ± 0.03)

Manus with five digits: digit I claw reduced, wide; digits II–V claws short, blunt; ungual tufts long, surpass digit tips. Palmar surface with five pads: thenar rounded, wide; hypothenar similar shape, larger; interdigital pads small, rounded; space between pads granular. Hind foot 25–31 mm, whitish, faint darker hairs on distal metatarsals; ungual tufts exceed claws. Plantar surface with six pads: four interdigital pads similar in size/shape; hypothenar pad ∼$ \frac{1}{4} $ size of thenar pad; plantar skin between pads granular (Fig. 3L).

**Figure 13:**
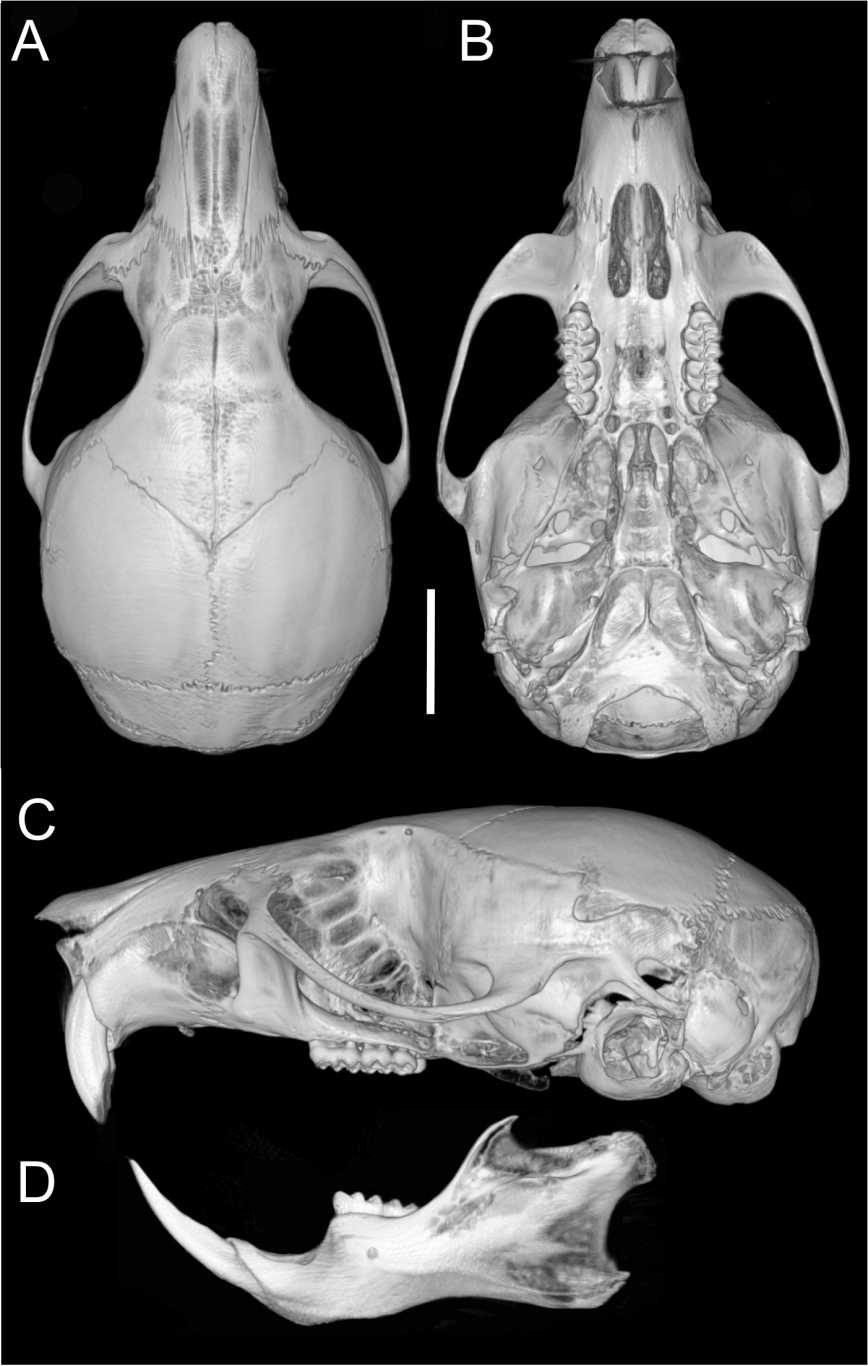
Cranium and mandible. Cranium in dorsal (A), ventral (B), and lateral (C) views, and mandible in labial view (D) of *Oreoryzomys jumandi* sp. nov. (MECN 8278, holotype; Estación Cientíûca Yanayacu, Napo, Ecuador). Scale = five mm. 3D reconstruction by C Koch and J Brito.

Skull (Figs. 6E–6F, 9E–9F, 13) small (CIL 21.02–23.49 mm), rounded cranial profile, no temporal ridges (Figs. 13A, 13C). Rostrum narrow (BR/ZB 11% ± 1.1), slightly elongated. Posterior nasal margin surpasses lacrimals; shallow zygomatic notch. Interorbital region wide; fronto-parietal suture V-shaped (Fig. 6E). Cranial box wide, rounded; interparietal wide. Lateral view (Fig. 13B): nasals surpass anterior face of incisors; gnatic process reduced. Postglenoid foramen subequal to subsquamosal fenestra; hamular process thin, lies distally over mastoid capsule (Fig. 9F). Alisphenoid strut absent (buccinator–masticatory foramen and accessory foramen ovale confluent). Stapedial foramen, squamosal–alisphenoid groove, sphenofrontal foramen present (carotid pattern 1, Voss, 1988). Incisive foramina short, not reaching M1 root, widest posteriorly (Fig. 6F). Posterolateral palatal pits large, recessed in shallow fossae. Mesopterygoid fossa extends anteriorly between maxillae, not between molar rows; bony roof perforated by short sphenopalatine vacuities. Middle lacerate foramen wide; auditory bulla distant from alisphenoid. Auditory bullae small, slightly inflated; stapedial process long, extends beyond alisphenoid edge (Fig. 9F). Capsular process of lower incisor alveolus below coronoid base; superior/inferior masseteric ridges converge anteriorly below m1; angular process short, does not reach condylar process; angular notch shallow.

Upper incisors with orange front enamel. M1 with anteromedian flexus dividing procingulum into subequal conules; anteroloph developed, attached to anterolabial conule by enamel bridge (Fig. 10C); paracone and protocone joined by enamel bridge; mesoloph long, narrow, joined to metacone by thin enamel bridge; M2 with shallow protoflexus; hypoflexus and metaflexus not interpenetrated; M3 without posteroloph; hypoflexus typically very shallow or infolded to form a lake; M1 three-rooted (no accessory labial root); M2 and M3 two-rooted; m1 with shallow anteromedian flexid; distinct anterolophid; metaconid connected to anterolophid by thin enamel bridge; short mesolophid fused to entoconid; m2 with anterolophid and long mesolophid fused to mesostylid (Fig. 11C); m3 small, slightly square; hypoflexid long, not reaching medial fossette; lower molars two-rooted.

**Comparisons:** *Oreoryzomys jumandi* sp. nov. differs in several morphological traits from *O. balneator* and *O. hesperus* (Table 4).

**Figure 14:**
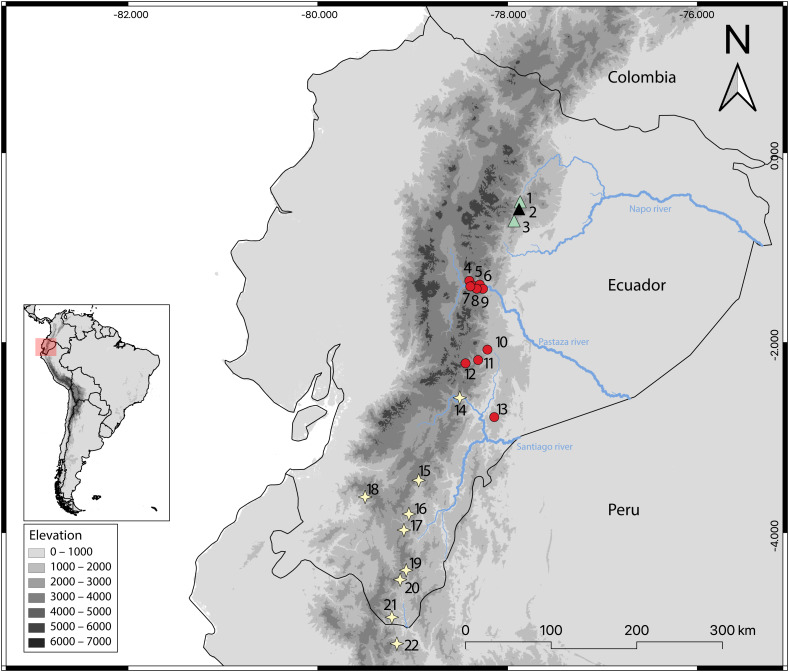
Geographic distribution of *Oreoryzomys* species in Ecuador and Peru. References: *O. balneator* = circles; *O. hesperus* = stars; *O. jumandi* sp. nov. = triangles. The black triangle indicates the type locality. Localites: **Ecuador, Napo:** 1 = Hosteria the Magic Roudabout (−0.516396, −77.867); 2 = Yanayacu Biological Station (−0.60077, −77.8883); 3 = Sierra Azul (−0.668056, −77.897222); **Tungurahua:** 4 = Reserva Vizcaya (−1.349582, −78.402425); 5 = Guamag (−1.40127, −78.36338); 6 = Reserva Machay (−1.400916, −78.287901); 7 = Chamana Pamba (−1.4238, −78.32541); 8 = Cerro Candelaria (−1.434161, −78.306616); 9 = La Palmera (−1.43386, −78.2587); **Morona Santiago:**10 = Sardinayacu (−2.074306, −78.211833); 11 = 9 de Octubre (−2.183949, −78.310135); 12 = Tinguichaca (−2.218628, −78.442569); 13 = Kutukú (−2.787306, −78.131583); **Azuay:** 14 = Arenales (−2.580824, −78.503564); **Zamora Chinchipe:** 15 = Yacuambi (−3.44991, −78.9366); 16 = El Denuncio (−3.80401, −79.0403); 17 = Sabanilla (−3.97253, −79.0924); **El Oro:** 18 = Chivaturco (−3.625, −79.501111); **Zamora Chinchipe:** 19 = Numbala Alto (−4.395008, −79.067896); 20 = Reserva Tapichalaca (−4.492083, −79.129778); 21 = Los Rubíes (−4.889, −79.2193); **Peru, Cajamarca:** 22 = Santuario Nacional Tabaconas-Namballe (−5.1666, −79.1666).

**Distribution:** The examined specimens of *Oreoryzomys jumandi* sp. nov. document a geographic range restricted to northeastern Ecuador (Province of Napo; see Fig. 14). Recorded elevations range from 1,980 to 2,500 m.

**Figure 15:**
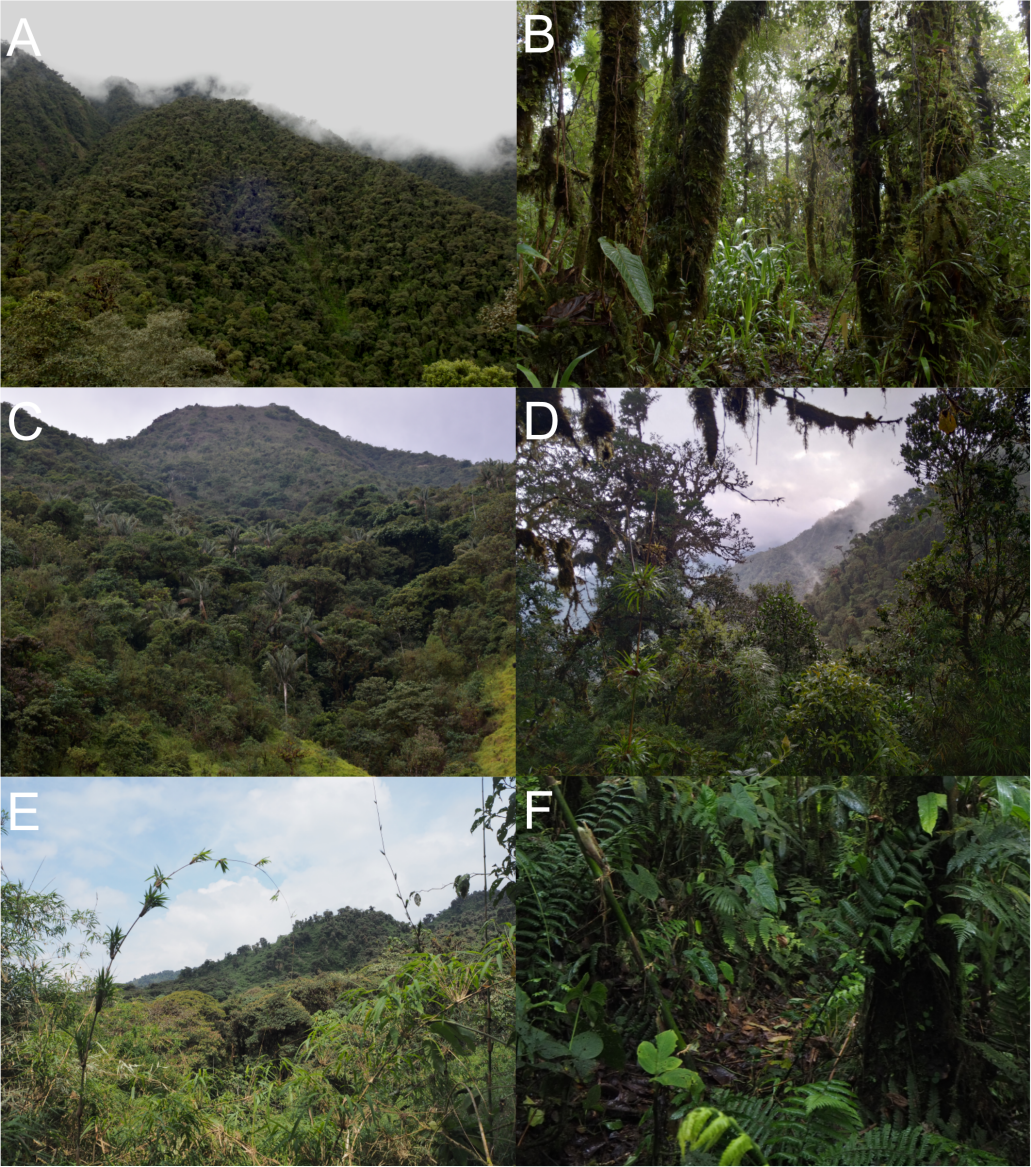
Habitat views of the three *Oreoryzomys* species. (A) Representative habitat of *O. balneator*; (B) understory detail from the Vizcaya Reserve, Tungurahua, Ecuador. (C) Habitat of *O. hesperus* in the Cordillera de Chilla, El Oro, Ecuador; (D) forest view in the Tapichalaca Biological Reserve, Zamora Chinchipe, Ecuador. (E–F) Habitat of *O. jumandi* sp. nov. at the Estación Científica Yanayacu, Napo, Ecuador. Photographs (A)–(C) by J Brito; (D) by N Tinoco; (E)–(F) by R Wistuba.

**Natural history:** *Oreoryzomys jumandi* sp. nov. occurs within a temperate zoogeographic zone (Albuja et al., 2012). Its habitat corresponds to montane forest (Ministerio del Ambiente del Ecuador, 2013), characterized by trees abundant in orchids, ferns, and bromeliads (Figs. 15E, 15F). Specimens of *O. jumandi* sp. nov. were collected in a mosaic of primary cloud forest, secondary forest, abandoned grasslands, and bamboo, with primary forest being the most dominant habitat. The terrain is steep and intersected by small streams. The understory is visually dominated by herbaceous families such as Poaceae (notably *Chusquea* spp.), Araceae, and Melastomataceae. *Oreoryzomys jumandi* sp. nov. was found in sympatry with *Microryzomys minutus*. Most individuals were captured at ground level, with only one specimen found on a fallen tree trunk.

**Specimens examined (*n* = 33): Ecuador: Napo**, Sierra Azul (MECN 945, QCAZ 7676–77), Yanayacu (MECN 8277–79, QCAZ 4993–94, 1592), Hostería the Magic Roundabout (QCAZ 7700–01, 7711–12, 7714–15, 7730–31, 7733–34, 7736, 7738–39, 7741–42, 7748, 7751–53, 7756, 7768, 7770, 7772, 7778).

## Discussion

### Generic uniqueness of *Oreoryzomys*

By the late 1980s, two monographs marked the beginning of the modern era in sigmodontine taxonomy. Both laid the groundwork for what would become, over the next 35 years, a major shift in our understanding of this diverse radiation. One was the revision of the Ichthyomyini (Voss, 1988); the other, the comprehensive reassessment and elevation of *Microryzomys* to generic rank (Carleton & Musser, 1989). The latter proved especially influential, catalyzing the near-complete reorganization of *Oryzomys*, which at the time was considered one of the more taxonomically difficult groups of South American rodents. This process, carried out through a series of pivotal studies (*e.g.*, Musser et al., 1998; Percequillo, 1998; Percequillo, 2003; Patton, da Silva & Malcolm, 2000; Bonvicino & Moreira, 2001; Voss, Gómez-Laverde & Pacheco, 2002; Weksler, 2003; Weksler, 2006), eventually led to the disaggregation of *Oryzomys* into ten distinct genera, including *Oreoryzomys* (Weksler, Percequillo & Voss, 2006).

Although Carleton & Musser (1989) discussed the taxonomic history of the so-called “pygmy forms of *Oryzomys*,” they made no mention of *balneator*. Even so, they later acknowledged the species’ morphological affinities with members of *Microryzomys*. As they noted (Musser & Carleton, 2005), *balneator* shares several key features with that genus, including small body size, soft fur, a long and sparsely haired tail, a delicate cranium with a rounded braincase and small bullae, and molars with similar brachyodont and pentalophodont patterns. The distinctiveness of *balneator* was further emphasized by Musser et al. (1998), who described it as a small-bodied, long-tailed species that was morphologically unlike other members of *Oryzomys*. Its possible association with *Microryzomys*, *Neacomys*, and *Oligoryzomys* began to be explored with the first molecular data for the species (Weksler, 2003). This association was later confirmed by Weksler (2006)), whose combined analysis of morphological and genetic data placed *balneator* as sister to *Microryzomys*. That relationship has since been recovered in multiple studies (*e.g.*, Hanson & Bradley, 2008; Brito et al., 2020; Percequillo et al., 2021).

Despite clear similarities, Musser & Carleton (2005:310) explained their decision not to include *balneator* in *Microryzomys*: “Although cognizant of such similarities when composing the *Microryzomys* revision (Carleton & Musser, 1989), equally numerous differences persuaded us not to include *balneator* within the genus. The cranium of *balneator* is larger and differently proportioned than both *M. altissimus* and *M. minutus*; its interorbital region is much broader and bears a slight postorbital shelf; the zygomata are anteriorly convergent, not squared; incisive foramina are exceptionally short, terminating well anterior to the M1s; the anteroconid is a single cone, not bifurcated as in *Microryzomys*; the hind feet are comparatively large and more elongate over the metatarsal region, digit V is short, and the plantar pad configuration is correspondingly altered.”

When *Oreoryzomys* was described, Weksler, Percequillo & Voss (2006) revisited the phenetic similarities with *Microryzomys* and outlined a set of distinguishing traits. These include countershaded pelage (*versus* the more uniformly colored fur of *Microryzomys*), differences in pedal morphology—such as the extent of claw V—and tail coloration, which in *Oreoryzomys* is unicolored or weakly bicolored only at the base. Cranial differences include the relative positions of the premaxillae and nasals, a more caudally oriented foramen magnum, and an undivided anteroconid on m1.

Although the taxonomic and morphological diversity of *Oreoryzomys* has increased from one to three species (this paper), many of the original diagnostic traits remain valid. Some, however, require more nuanced interpretation. For example, while the incisive foramina are clearly short in *balneator*, this is not the case in *jumandi*. Similarly, the anteroconid (or procingulum) of m1, although formed by two conulids, appears undivided in adult specimens due to conulid fusion. Despite these and other minor differences, the morphological distinctiveness of *Oreoryzomys* at the generic level remains well supported.

**Figure 16:**
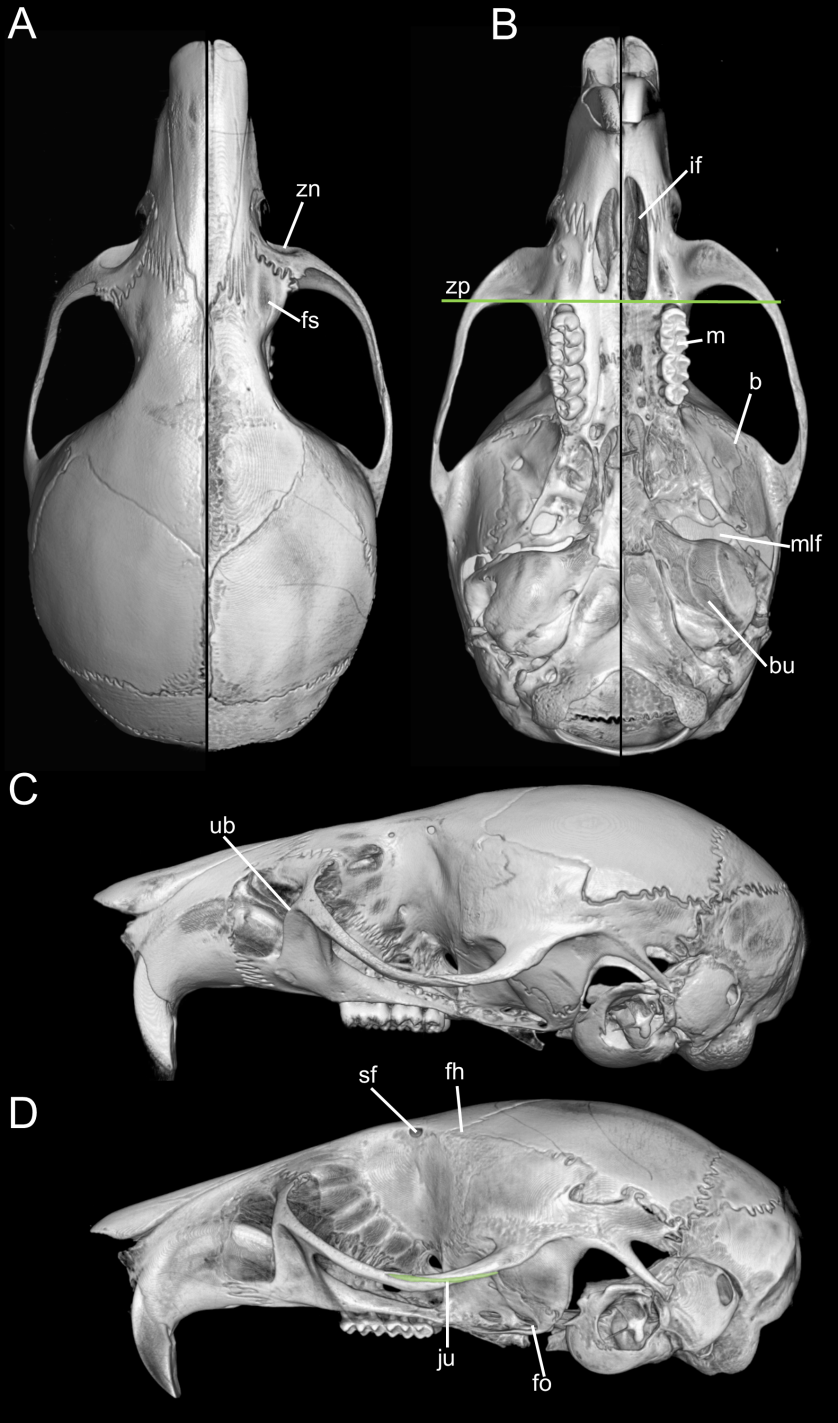
Composite image illustrating key cranial differences between *Oreoryzomys balneator* (MECN 5795; left half of cranium: A–B; lateral view: (C) and *Microryzomys altissimus* (MECN 7197; right half of cranium: A–B; lateral view: (D). Abbreviations: b, braincase; bu, bulla; fh, frontal “horn;” fo, foramen ovale; fs, frontal sinus; if, incisive foramen; ju, jugal; m, molar row; mlf, middle lacerate foramen; sf, supraorbital foramen; ub, upper border of zygomatic plate; zn, zygomatic notch; zp, “zygomatic plane” defined by the posterior margin of the zygomatic plate. Three-dimensional reconstructions by C Koch and J Brito.

Traits originally highlighted by Musser & Carleton (2005) and Weksler, Percequillo & Voss (2006) continue to justify its recognition as separate from *Microryzomys*. Some of the clearest differences relate to cranial architecture. Although both genera may appear superficially similar in profile, *Microryzomys* has a more protruded rostrum, and its zygomatic plate lies well anterior to the plane of the M1s. In addition, the zygomatic plate in *Microryzomys* is narrower and lacks a pronounced dorsal notch, and the braincase is more rounded and expanded, with frontal “horns” projecting over the interorbital region. One of the most pronounced differences lies in molar size. *Microryzomys* exhibits microdonty (sensu Schmidt-Kittler, 2006), with reduced molars that do not correspond to a shorter palate but rather reflect an anterior displacement of the basicranium. This shift affects associated structures, such as the parapterygoid plate (which is shortened and broadened), the middle lacerate foramen (enlarged), and the ectotympanic bone (reduced; Fig. 16).

Taken together, these cranial, dental, and external differences continue to support the recognition of *Oreoryzomys* as a distinct genus under current systematic frameworks (*e.g.*, Weksler, Percequillo & Voss, 2006; Brito & Pardiñas, 2025). Still, the morphological and genetic similarities between *Oreoryzomys* and *Microryzomys* remain considerable. Their close relationship is consistently supported by unilocus and multilocus phylogenies, with a divergence time estimated at approximately 2 million years ago (Percequillo et al., 2021). In this context, shared traits are best interpreted as the result of shared ancestry, rather than convergent evolution.

Overall, *Oreoryzomys* and *Microryzomys* likely represent the products of parapatric divergence shaped by Andean environmental dynamics (Patton, Myers & Smith, 1990). Both are small-bodied, long-tailed oryzomyines, but while *Oreoryzomys* appears specialized for cool, humid, mid-elevation Andean forests, *Microryzomys* is more commonly associated with drier, higher-elevation páramo habitats (Carleton & Musser, 1989).

### Rethinking *Oreoryzomys*

*Oreoryzomys* was established nearly two decades ago in the context of dismantling the polytypic genus *Oryzomys*. This restructuring was the culmination of successive genetic and morphological reevaluations within the tribe Oryzomyini (*e.g.*, Musser et al., 1998; Weksler, 2003; Weksler, 2006; Weksler, Percequillo & Voss, 2006). Published alongside nine other genera—*Aegialomys*, *Cerradomys*, *Eremoryzomys*, *Euryoryzomys*, *Hylaeamys*, *Mindomys*, *Nephelomys*, *Sooretamys*, and *Transandinomys*—*Oreoryzomys* was introduced within a large-scale taxonomic revision. Possibly due to this “massive” presentation, its diagnosis resembles a morphological description more than a classical, concise diagnosis (*i.e., character essentialis*; cf. Mayr, Linsley & Usinger, 1953:155). Indeed, the simultaneous description of ten genera in a single contribution is exceptional in the history of sigmodontine rodent taxonomy. Comparable instances can only be found in the earliest taxonomic efforts (*e.g.*, Waterhouse, 1837; see D’Elía & Pardiñas, 2007). Given the comparative nature of presenting these genera—many of which share morphological traits—it appears that Weksler, Percequillo & Voss (2006) opted for morphological descriptions (*character naturalis*; cf. Mayr, Linsley & Usinger, 1953:155) as a more practical form of diagnosis.

The core issue with using detailed morphological descriptions as diagnoses is that such diagnoses inherently lack predictive power—that is, the ability to accommodate new species without requiring modification. This limitation is evident in the case of *Oreoryzomys*, whose original diagnosis coincided with its monotypic status. At the time, the genus contained only a single species, *O. balneator*, and the diagnosis was effectively a detailed description of that species. In fact, the currently available diagnosis includes specific and individually variable features such as dorsal and ventral coloration patterns (including gular and pectoral patches; cf. Weksler, Percequillo & Voss, 2006:21).

Diagnosis and description serve different functions. According to (Mayr, Linsley & Usinger, 1953:155) “The diagnosis serves to distinguish the species (or whatever taxon is involved) from other known similar or closely related ones. The general description…should present a general picture of the described taxon... not only on characters that are diagnostic…but also characters that may distinguish the species from yet unknown species... also provide information that may be of interest to others besides taxonomists.”. For the purposes of this contribution, it is evident that the approach taken by Weksler, Percequillo & Voss (2006) reflects an evolution in thinking that spans several years. For example, when revising the Ichthyomyini, Voss (1988) provided classical, concise diagnoses comprising selected traits, followed by separate detailed descriptions. This approach was maintained in subsequent works during the 1990s (*e.g.*, revision of *Lundomys*; Voss & Carleton, 1993). However, in the description of *Handleyomys*, Voss, Gómez-Laverde & Pacheco (2002) introduced a new format: a combined but explicitly labeled “morphological diagnosis and description.” This practice evolved further in Weksler, Percequillo & Voss (2006), where diagnosis and description were merged under the heading “morphological diagnosis”—a format that continues in recent works (*e.g.*, description of *Casiomys*; Voss, 2024).

Regardless of the significance—if any—of this shift in diagnostic style, the key point is the differing effectiveness of these two approaches in achieving their primary goal: aiding in the recognition of taxa (Simpson, 1945; Mayr, Linsley & Usinger, 1953). Concise diagnoses rely on an arbitrary but necessary decision: the a priori selection of diagnostic traits. In contrast, combined diagnoses/descriptions avoid this limitation by including all known morphological features. It almost goes without saying that the latter are more comprehensive and therefore could be interpreted as more effective as recognition tools. However, from an epistemological perspective, concise diagnoses represent “closed” lists—fixed sets of features—while combined descriptions are inherently “open” or infinite lists (cf. Eco, 2009), continually subject to revision as new characters are discovered. This makes the latter less stable: any addition to the morphological understanding of a taxon necessitates an emendation of the diagnosis. A further distinction lies in their practical application. When working with physical specimens, concise diagnoses are easier to follow and apply, whereas extensive character lists can be overwhelming and may hinder rather than help taxonomic work (Mayr, Linsley & Usinger, 1953).

In the specific case of *Oreoryzomys*, the original concise diagnosis by Thomas (1900) for *balneator* was later replaced by the combined approach used when the genus was formally recognized by Weksler, Percequillo & Voss (2006). As the genus was monotypic at the time, the combined diagnosis essentially described *O. balneator*. With the recognition of three species in the current classification, an emended generic diagnosis is now required, along with individual diagnoses for each species. Accordingly, a revised, classical-style diagnosis for the genus is provided below:

**Table:** 

Genus *Oreoryzomys*Weksler, Percequillo & Voss, 2006

Type species: *Oryzomys balneator* Thomas, 1900.

Contents (listed in chronological order): *Oreoryzomys balneator* (Thomas, 1900), *Oreoryzomys hesperus* (Anthony, 1924), and *Oreoryzomys jumandi* Brito, Vargas, García, Tinoco & Pardiñas, new species.

**Enmended diagnosis:** A genus of oryzomyine rodents characterized by the following combination of traits—size small (HBL 62–125 mm) with tail notably longer than head and body (117–138%); ear small; hind feet short and slender with six fleshy plantar pads including a distinct hypothenar pad; ungual tufts surpassing moderately developed pedal claws; skull having a short and pointed rostrum, hourglass-shaped and somewhat broad interorbit and large, and rounded braincase devoid of ridges; nasals surpassing anteriorly incisors and premaxillae; zygomatic notch inconspicuous; interparietal large; robust zygomatic arches slightly divergent backwards; jugal absent; zygomatic plate narrow and parallel sided; parietal with small lateral expansion; incisive foramina short (except *O. hesperus*) and broad; bony palate broad slightly extended beyond end of third molars with posterolateral palatal pits large and recessed in shallow fossae; alisphenoid strut absent; carotid circulation pattern 1; postglenoid foramen and subsquamosal fenestra large and subequal in size; periotic broadly exposed; capsular process of lower incisor conspicuous; semilunar notch inconspicuous; incisors narrow and slightly opisthodont; brachyodont molars small but not diminutive; upper first molar rectangular-shaped due to a broad procingulum composed of two subequal conules and separated by a persistent anteromedian flexus; anterolophs and mesolophs conspicuously developed in first and second upper molar; third upper molar small and triangular; main cusps of lower molars nearly opposite; first lower molar with metaconid appearing as an isolated cusp and anteromedian flexid scarcely persistent; 12 ribs; stomach unilocular-hemiglandular with minor extension of glandular epithelium into corpus; penis large and trifid (after Thomas, 1900; Anthony, 1924; Weksler, Percequillo & Voss, 2006; this article); vaginal–uterine portion with paired lateral lobes.

**Mountains of diversity (an Andean odyssey)**.—The Andes have played a fundamental role in the diversification and speciation of South American cricetid rodents, acting as a geographic barrier that promotes isolation and vicariance processes, while also generating a wide variety of habitats and ecological gradients. The vicariance–dispersal model, widely supported, explains lineage divergence in relation to orogenic events and environmental changes, as observed in genera such as *Transandinomys* (Weksler, Percequillo & Voss, 2006), *Pattonimus* (Brito et al., 2020), and *Cerradomys* (Oliveira da Silva et al., 2024). Allopatric speciation has been favored by prolonged isolation among populations in the different Andean mountain ranges, evidenced in species such as *Thomasomys pardignasi* (Brito et al., 2021a; Brito et al., 2021b) and *Punomys lemminus* (Quiroga-Carmona, Storz & D’Elía, 2023). Additionally, the ecological diversity and complex altitudinal gradients of the Andes have promoted parapatric or sympatric speciation, as seen in *Oligoryzomys* (Hurtado & D’Elía, 2022). The Pleistocene hypothesis proposes that glacial cycles fragmented Andean forests, driving genetic differentiation in numerous lineages (Patton, da Silva & Malcolm, 2000). Collectively, the Andes constitute a biodiversity hotspot and an apparently key driver in the evolution of Neotropical rodent fauna (Parada et al., 2013; Leite et al., 2014; Maestri & Patterson, 2016; Salazar-Bravo et al., 2023; Vallejos-Garrido et al., 2023).

With ancient sigmodontine fossil records virtually absent (see Ronez et al., 2023), most diversification estimates rely heavily on molecular phylogenies (*e.g.*, Ronez et al., 2021; Steppan, Adkins & Anderson, 2004; Steppan & Schenk, 2017; Maestri, Upham & Patterson, 2019). Needless to say, the accuracy of these inferences is highly sensitive to the extent of taxonomic sampling. In such a context, the revision of a single genus, *Oreoryzomys*, which resulted in a threefold increase in species number, allows for testing the aforementioned hypotheses. This issue becomes even more compelling in light of the recent discovery of more than 10 new taxa in less than a decade (see below).

Despite these limitations, there is strong support for a long-standing evolutionary association between the sigmodontine tribe Oryzomyini and what is now the Tropical Andes. Oryzomyini currently includes 174 species across 31 extant genera (according to Brito & Pardiñas, 2025, Table 1, and this paper), making it the most diverse tribe within the subfamily Sigmodontinae (see also Brito et al., 2020; Percequillo et al., 2021). Although some argue that this figure may be slightly inflated—two genera, *Scolomys* and *Zygodontomys*, are retained within the tribe despite their morphological and ecological distinctiveness (Voss, 1991; Patton & da Silva, 1995)—even excluding such cases, Oryzomyini still stands out due to its exceptional generic richness, nearly double that of the second most diverse tribe, Akodontini (Brito & Pardiñas, 2025). Furthermore, a substantial proportion of this oryzomyine diversity is concentrated in the Tropical Andes (Prado et al., 2015).

Importantly, the Andes are not only home to the highly speciose Oryzomyini, but also to significant components of other tribes such as Ichthyomyini, Neomicroxini, and Thomasomyini. These groups also show pronounced diversity in the northern Andean ranges (*e.g.*, Voss, 1988; Salazar-Bravo et al., 2023; Patton, Pardiñas & D’Elía, 2015), and are increasingly recognized for harboring substantial cryptic diversity (*e.g.*, Cañón et al., 2020; Brito et al., 2022a; Brito et al., 2022b; Salazar-Bravo et al., 2023; Pacheco & Ruelas, 2023).

Recent findings from Ecuador exemplify the magnitude of the taxonomic gap in the region. Even well-studied genera continue to yield new species. Voss (2003:39) was prescient in noting that “the middle elevations of the Cordillera Oriental remain a virtual terra incognita from the mammalogical perspective,” highlighting “an elevational band at least 2,000 m high and more than 1,500 km long from south to north that is currently unrepresented by any significant collections of mammals” (Voss, 2003:40). The upper Napo basin region, as part of the Eastern Cordillera, although little explored, is considered an area of high endemism (Lynch & Duellman, 1980). More recent surveys in the Quijos Valley have identified it also as a zone of high endemism (Cisneros-Heredia & Mcdiarmid, 2006; Guayasamin & Funk, 2009).

Fortunately, targeted fieldwork has begun to address this sampling gap. Intensive trapping campaigns have been carried out in previously undersampled regions along the eastern slope of the Andes, particularly within the geographic band highlighted by Voss (2003). These efforts often involved significant logistical challenges, including aerial surveys to identify access routes—such as in the Cordillera de Kutukú (Brito et al., 2021a; Brito et al., 2022b) and Cordillera del Cóndor (Brito et al., 2021b)—as well as repeated expeditions into areas characterized by high rainfall and humidity. Notable examples include surveys in the Valle de Cosanga (Lee Jr et al., 2006), Sumaco (Lee Jr et al., 2008), Reserva Tapichalaca (Lee Jr et al., 2023), Parque Nacional Sangay (Lee et al., 2011; Brito & Ojala-Barbour, 2016), and Parque Nacional Yacuri (Lee Jr et al., 2018). In addition, the detection of elusive taxa in these habitats has required the implementation of specialized sampling protocols (Brito & Ojala-Barbour, 2016).

In parallel, the strengthening of national capacities, particularly the development of robust biological collections, has been a central goal of institutions like the Instituto Nacional de Biodiversidad (INABIO, formerly Museo Ecuatoriano de Ciencias Naturales), which is now the Ecuadorian mammal collection with the largest number of holotypes (*n* = 15). These efforts have been supported by advances in local molecular laboratories, such as the Pontificia Universidad Católica del Ecuador (QCAZ) and INABIO, and classical morphological analysis (*e.g.*, comparative anatomy and morphometry), which allows for integrative taxonomic evaluations.

As a result of this multifaceted strategy—combining intensive fieldwork, specimen collection, applied technologies, and integrative systematic approaches—20 new taxa have been formally described. These include two new tribes (Neomicroxini and Rhagomyini; Pardiñas et al., 2021; Pardiñas et al., 2022), one new genus (*Pattonimus*; Brito et al., 2020), and 17 new species. This dramatic increase in recognized taxonomic diversity is not unique to Ecuador. Over the same period, numerous new taxa have been described from Colombia and Peru, encompassing new species and subspecies (*e.g.*, Pacheco & Ruelas, 2023; Colmenares-Pinzon, 2021; Ruelas et al., 2021), as well as at least one additional genus, *Incanomys* (Zeballos et al., 2025). On the other hand, preliminary surveys in historically underexplored regions, such as Abiseo in northern Peru, continue to reveal high levels of previously undocumented diversity (*e.g.*, Pavan et al., 2024; Pavan et al., 2025).

Many cricetid genera primarily or secondarily associated with the northern Andes remain totally or partially unrevised, including taxa emblematic such as *Aepeomys*, *Microryzomys*, and *Neomicroxus* (Carleton & Musser, 1989; Pacheco, 2015; Cañón et al., 2020). Even in well-sampled groups, cryptic diversity persists, as shown by recent studies of *Nephelomys* (*e.g.*, Ruelas et al., 2021). Only rarely do taxonomic revisions result in a potential net reduction in recognized diversity (*e.g.*, Brito & Pardiñas, 2025).

Ultimately, the Tropical Andes present a crucial paradox: the race to describe new species is running against the pace of environmental degradation and biodiversity loss. Given the recurring economic crises in Andean countries and the increasing pressure to exploit natural resources (*e.g.*, Cogălniceanu & Szekely, 2025), it is essential to integrate taxonomic and biodiversity survey programs into national policy agendas as a strategic measure to mitigate irreversible ecological losses. In this broader biogeographical and conservation framework, the revised taxonomy of *Oreoryzomys* not only clarifies the genus’s evolutionary identity but also exemplifies the latent diversity still awaiting discovery in the Tropical Andes.

## Conclusions

An integrative systematic revision, based on extensive field sampling and comprehensive examination of museum specimens, demonstrates that the little-known *Oreoryzomys balneator* comprises a complex of distinct species. As a result, *O. balneator* is redescribed, *O. hesperus* is elevated to full species status, and a new species is formally described, all representing Ecuadorian populations inhabiting montane forests on the slopes of the Andes. Future studies require increasing collections in areas with a lack of information, such as the eastern border between Ecuador and Colombia and northwestern Peru.

##  Supplemental Information

10.7717/peerj.20515/supp-1 Supplemental Information 1 Bayesian inference phylogenetic tree based on the Cytb gene matrix The tree is unedited.

10.7717/peerj.20515/supp-2 Supplemental Information 2 Bayesian inference phylogenetic tree based on the IRBP gene matrix The tree is unedited.

10.7717/peerj.20515/supp-3 Supplemental Information 3 Ultra-fast bootstrap (boot) phylogenetic tree based on the Cytb gene matrix The tree is unedited.

10.7717/peerj.20515/supp-4 Supplemental Information 4 Ultra-fast bootstrap (boot) phylogenetic tree based on the IRBP gene matrix The tree is unedited.

## Additional Information and Declarations

### Field Study Permissions

The following information was supplied relating to field study approvals (*i.e.*, approving body and any reference numbers): Field experiments were approved by the Ministerio del Ambiente, Agua y Transición Ecológica del Ecuador.

### DNA Deposition

The following information was supplied regarding the deposition of DNA sequences: The Cytb and IRBP sequences are available at GenBank: PV962869–PV962900 and PV962857–PV962868.

### Data Availability

The following information was supplied regarding data availability: The reconstructed CT data of the species *Oreoryzomys jumandi* sp. nov. (MECN 8278, holotype); *Oreoryzomys hesperus* (MECN 4789); *Oreoryzomys balneator* (MECN 5009), and *Oreoryzomys balneator* (MECN 5795), are available at MorphoBank: https://doi.org/10.7934/P5998.

### New Species Registration

The following information was supplied regarding the registration of a newly described species: Publication LSID: urn:lsid:zoobank.org:pub:5E61A4AC-BF4E-413C-B2D6-E9139759D6E3 Oreoryzomys jumandi sp. npv. LSID: urn:lsid:zoobank.org:act:86B4AE13-FF63-437E-89E8-516B1BE96F0F.
