# Supplementary figures and images for: Mountains of diversity: a systematic revision of the Andean rodent genus Oreoryzomys (Cricetidae: Sigmodontinae)
Source: PeerJ. 2026 Jan 9;14:e20515. doi: 10.7717/peerj.20515 (PMC12794643; doi:10.7717/peerj.20515)

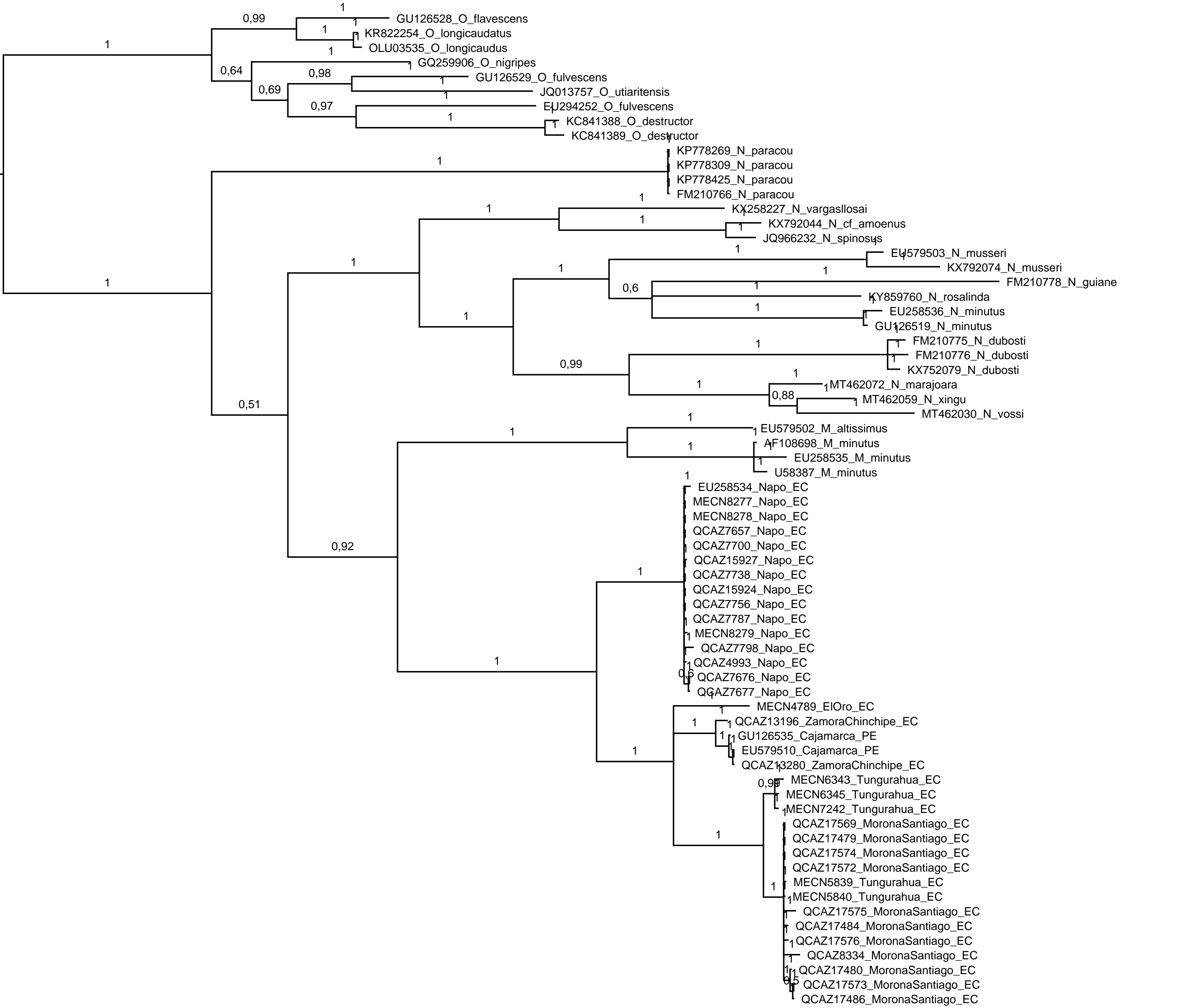

0.05

Supplement: Supplemental Information 1 — The tree is unedited. [file peerj-14-20515-s001.pdf]

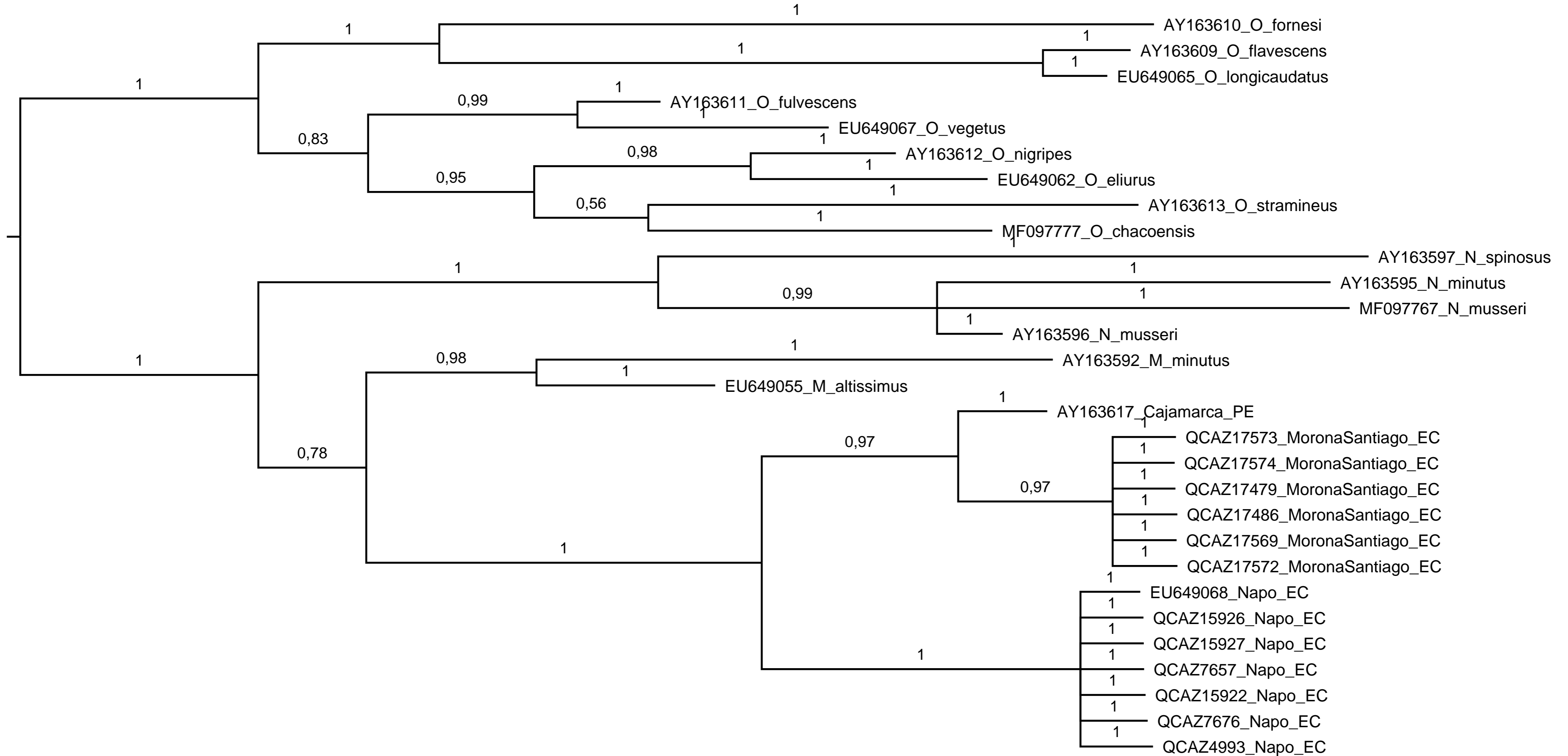

Supplement: Supplemental Information 2 — The tree is unedited. [file peerj-14-20515-s002.pdf]

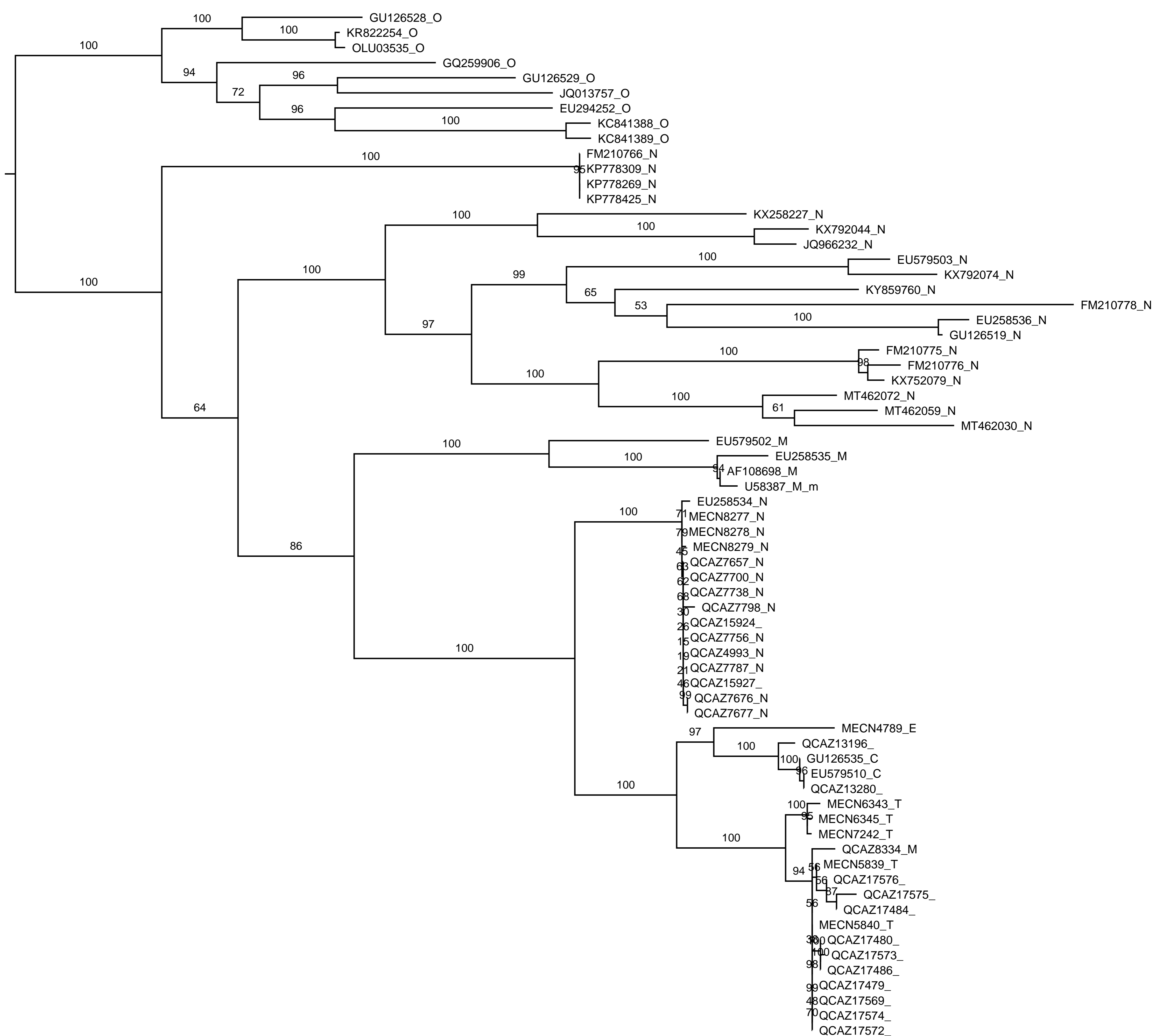

Supplement: Supplemental Information 3 — The tree is unedited. [file peerj-14-20515-s003.pdf]

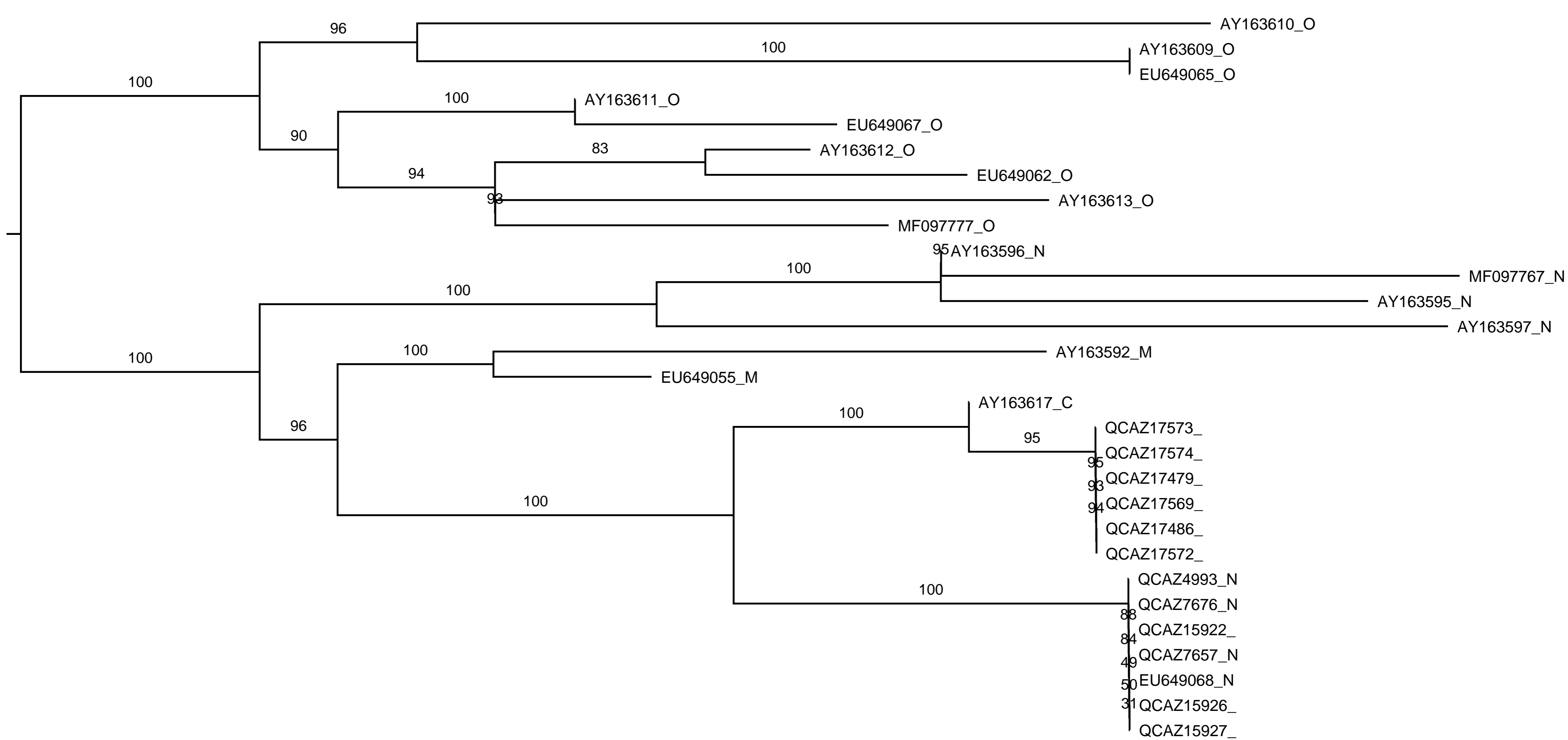

0.002

Supplement: Supplemental Information 4 — The tree is unedited. [file peerj-14-20515-s004.pdf]
